# Sensitivity to missing not at random dropout in clinical trials: Use and interpretation of the trimmed means estimator

**DOI:** 10.1002/sim.9299

**Published:** 2022-01-31

**Authors:** Audinga‐Dea Hazewinkel, Jack Bowden, Kaitlin H. Wade, Tom Palmer, Nicola J. Wiles, Kate Tilling

**Affiliations:** ^1^ Population Health Sciences, Bristol Medical School University of Bristol Bristol UK; ^2^ Medical Research Council Integrative Epidemiology Unit, Bristol Medical School University of Bristol Bristol UK; ^3^ Exeter Diabetes Group (ExCEED), College of Medicine and Health University of Exeter Exeter UK; ^4^ Centre for Academic Mental Health, Population Health Sciences, Bristol Medical School University of Bristol Bristol UK

**Keywords:** bias quantification, dropout, missing not at random, randomized controlled trials, sensitivity analyses, trimmed means

## Abstract

Outcome values in randomized controlled trials (RCTs) may be missing not at random (MNAR), if patients with extreme outcome values are more likely to drop out (eg, due to perceived ineffectiveness of treatment, or adverse effects). In such scenarios, estimates from complete case analysis (CCA) and multiple imputation (MI) will be biased. We investigate the use of the trimmed means (TM) estimator for the case of univariable missingness in one continuous outcome. The TM estimator operates by setting missing values to the most extreme value, and then “trimming” away equal fractions of both groups, estimating the treatment effect using the remaining data. The TM estimator relies on two assumptions, which we term the “strong MNAR” and “location shift” assumptions. We derive formulae for the TM estimator bias resulting from the violation of these assumptions for normally distributed outcomes. We propose an adjusted TM estimator, which relaxes the location shift assumption and detail how our bias formulae can be used to establish the direction of bias of CCA and TM estimates, to inform sensitivity analyses. The TM approach is illustrated in a sensitivity analysis of the CoBalT RCT of cognitive behavioral therapy (CBT) in 469 individuals with 46 months follow‐up. Results were consistent with a beneficial CBT treatment effect, with MI estimates closer to the null and TM estimates further from the null than the CCA estimate. We propose using the TM estimator as a sensitivity analysis for data where extreme outcome value dropout is plausible.

## 1 INTRODUCTION

Randomized controlled trials (RCT) are considered the gold standard for assessing causality, because randomization balances observed and unobserved variables across the treatment groups, on average. However, RCTs remain vulnerable to other sources of bias, including from missing data due to dropout. In this article, we consider the case of an RCT with an incomplete continuous outcome, measured at a single time‐point.

The impact of missing data depends on the missingness mechanism and the analysis model. Three missingness mechanisms can be distinguished: missing completely at random (MCAR), missing at random (MAR), and missing not at random (MNAR). With MCAR, missingness is unrelated to any measured or unmeasured characteristics and the observed sample is a representative subset of the unobserved full data. MAR means the missingness can be explained by observed data and, with MNAR, missingness is a function of the unobserved data (in our case, the outcome) itself.^1^, ^2^, ^3^

There are four general ways of dealing with incomplete data: a complete case analysis (CCA), inverse probability weighting (IPW), multiple imputation (MI), and maximum likelihood (ML) based inference. A CCA is the analysis model intended to be applied to the trial data at its outset, restricted only to individuals with observed outcomes. Assuming correct model specification, CCA in our scenario is unbiased if the outcome is MCAR or MAR, conditional on the covariates in the analysis model.^4^, ^5^, ^6^ With IPW, the CCA model is fitted, but each individual is now weighted by the inverse of its probability of being observed.^7^, ^8^ While CCA and IPW discard any incomplete cases, ML and MI use all available information. With ML, parameters are estimated by maximizing the probability density function (the likelihood) of the observed data, with missing data values removed from the likelihood through a process of summation or integration.^1^, ^2^, ^9^ With MI, the observed data are used to repeatedly predict—“impute”—the missing values. MAR can be intuitively understood as the assumption that the distributions of the missing and observed values (here, outcome values) are the same given the fully observed variables.^6^ In MI, this distribution is estimated, conditional on the observed model covariates and optional auxiliary variables. The uncertainty in the imputation process is dealt with by generating multiple complete datasets, to which the analysis model is applied,^2^, ^10^, ^11^, ^12^ with the resulting estimates pooled using Rubin's rules.^10^

Broadly, IPW, MI, and ML will be valid if the models are correctly specified and if the data are MAR, conditional on the covariates in the IPW model, imputation model, and analysis model, respectively. When data are MNAR, however, the treatment effect estimate will be biased in general for any of these approaches.^2^, ^3^, ^4^ Observed data cannot be used to determine whether data are MAR or MNAR, and, consequently, if estimates from CCA, MI, ML, or IPW analyses are likely to be biased. Guidelines published by the National Research Council (US) and the European Medical Agency strongly recommend examining sensitivity to departures from the missing data assumptions made in the primary analysis.^3^, ^13^, ^14^ Two approaches for dealing with incomplete data, which can be adapted to sensitivity analyses, are selection models (SM) and pattern mixture models (PMM). SM specify the relationship between outcome and the probability of being missing, while PMMs define the outcome distribution for unobserved individuals across a range of missingness patterns.^2^ Such approaches require the specification of a missingness model or numerical sensitivity parameters, to characterize the unknown missingness mechanism. Choosing plausible distributions and values, however, is often challenging, requiring elicitation of expert opinion.^15^, ^16^, ^17^ We propose using the trimmed means (TM) estimator in addition or as a simpler alternative to SM and PMM sensitivity analyses.

Permutt and Li^18^ suggested a TM estimator for RCTs where patients who drop out have more extreme (unobserved) outcome values than those who do not drop out (eg, lower value dropout, when comparator group patients, not experiencing a benefit of treatment, leave the study early). Observations are ordered within each treatment group, with the missing observations assigned the lowest rank. Equal proportions of data are trimmed away from the lower end of both treatment group distributions and the TM treatment effect estimate is obtained from a linear regression of the exposure and outcome variables, using the remaining “trimmed” data. The TM estimator requires the specification of only a single sensitivity parameter—the size of the trimming fraction, which is bounded from below by the highest observed proportion of dropout.

The TM estimator will give an unbiased estimate of the true treatment effect, given two main assumptions—the location shift assumption and the strong MNAR assumption. The first specifies identical distributions of the outcome in the treatment groups, with the only difference being a mean shift. The second restricts all dropout to the fraction that is trimmed away.^19^, ^20^ Previous studies have performed a variety of simulations investigating the type 1 error and power of the TM estimator across a range of clinical scenarios^20^ and for various MNAR/MAR dropout patterns.^19^ However, the biases that arise from violations of the strict TM assumptions and how to correct for these biases have yet to be established.

In this article, we derive formulae for the bias resulting from the violation of the location shift and strong MNAR assumptions for normally distributed outcomes, and illustrate the theoretical results with simple simulations of various MNAR/MAR mechanisms. Additionally, we propose an adjustment to the estimator which relaxes the location shift assumption and leaves the estimator reliant only on the strong MNAR assumption and normality. For the purpose of this article, consistent with previous publications,^18^, ^19^, ^20^ we primarily consider worst value dropout. The principle, however, is equally applicable to higher value dropout, which may occur when patients leave the study perceiving themselves to be recovered, or when higher values indicate a worse response. We also primarily consider the case of 50% fixed trimming, but provide more general formulae for alternate trimming fractions. The TM approach is illustrated in an application to the CoBalT RCT (registration ISRCTN38231611), which compares the effectiveness of cognitive behavioral therapy (CBT) as an adjunct to pharmacotherapy vs usual care in patients with treatment resistant depression. We use the TM estimator and two MI models in a sensitivity analysis of the long‐term CCA treatment effect, estimated at 46 months. We have developed an R package “tmsens” for performing a TM regression and conducting a TM estimator sensitivity analysis, available from https://github.com/dea‐hazewinkel/tmsens.

## 2 METHODS

### 2.1 Notation

Consider a clinical trial with *n* subjects, with a continuous outcome, *Y*, with patients randomized to receive an active treatment (R=1) or comparator (R=0). Let $y_{ij}$ denote the observed outcome for patient *i* randomized to treatment group *j*, where $i=1,\ldots ,n_{j}$ and j=0,1. Let $\mu_{j}$ be the population mean for arm *j*, with $\mu_{j}=𝔼[Y|R=j]$, and its estimate, ${\hat{\mu }}_{j}$, obtained from the corresponding sample mean:

$${\hat{\mu }}_{j}=\frac{1}{n_{j}}\overset{n_{j}}{\underset{i=1}{\sum }}\;y_{ij}.$$

When there are no missing outcomes in the trial, each ${\hat{\mu }}_{j}$ is an unbiased estimate of $\mu_{j}$. Let β denote the true treatment effect, given by the difference in treatment group means:

$$\beta =\mu_{1}-\mu_{0},$$

where β is estimated by $\hat{\beta }$:

$$\hat{\beta }={\hat{\mu }}_{1}-{\hat{\mu }}_{0}.$$

When the trial outcome is incomplete, we define a missing indicator *M*, which equals 0 if *Y* is observed and 1 if *Y* is not observed. Let ${\hat{\mu }}_{cj}$ be the mean of all observed values for group *j*, given by ${\hat{\mu }}_{cj}=\frac{1}{n_{mj}}\sum_{i=1}^{n_{j}}y_{ij}(1-m_{ij})$, with $n_{mj}=\sum_{i=1}^{n_{j}}(1-m_{ij})$ and $m_{ij}$ being subject *i*'s missingness indicator. Then ${\hat{\mu }}_{cj}$ estimates the population complete case mean $\mu_{cj}=𝔼[Y|R=j,M=0]$ and the complete case estimand of the treatment effect is given by

$$\beta_{c}=\mu_{c1}-\mu_{c0},$$

with $\beta_{c}$ estimated by

$${\hat{\beta }}_{c}={\hat{\mu }}_{c1}-{\hat{\mu }}_{c0}=\frac{1}{n_{m1}}\overset{n_{1}}{\underset{i=1}{\sum }}\;y_{i1}(1-m_{i1})-\frac{1}{n_{m0}}\overset{n_{0}}{\underset{i=1}{\sum }}\;y_{i0}(1-m_{i0}).$$

### 2.2 TM estimator

We first define the population trimmed mean, $\mu_{tj}$, in the absence of dropout ($m_{ij}=0$ ∀ *i*). For treatment group *j*, $\mu_{tj}$ is given by the expected value of all observations exceeding the quantile $F^{-1}(p)$:

(1) $$\mu_{tj}=𝔼[Y|R=j,Y>F^{-1}(p)],$$

where *p* is the proportion of outcomes trimmed away from the lower end of the distribution of each group *j* (eg, for p=0.25, the bottom 25% of the distribution would be removed). Then $\mu_{tj}$ is estimated by

(2) $${\hat{\mu }}_{tj}=\frac{1}{n_{tj}}\overset{n_{j}}{\underset{y_{ij}>{\hat{F}}^{-1}(p)}{\sum }}\;y_{ij},$$

with $n_{tj}$ being the sample size after trimming ($n_{tj}=\left\lceil n_{j}(1-p)\right\rceil$, with ⌈⌉ the ceiling function).

Let $\beta_{t}$ denote the TM effect estimand, given by the difference in population trimmed means ($\beta_{t}=\mu_{t1}-\mu_{t0}$), estimated by $\hat{\beta_{t}}={\hat{\mu }}_{t1}-{\hat{\mu }}_{t0}$. If the outcomes within each treatment group are normally distributed with underlying mean $\mu_{j}$ and variance $\sigma_{j}^{2}$, then $\mu_{tj}=𝔼[Y|R=j,Y\geq \sigma_{j}\Phi^{-1}(p)+\mu_{j}]$. If the treatment group SDs are equal ($\sigma_{1}=\sigma_{0}$), $C=\sigma_{j}\Phi^{-1}(p)$ is common across treatment groups and the population TM effect, $\beta_{t}$, is identical to the population mean difference β, since

(3) $$\begin{array}{ll} \beta_{t} & =𝔼[Y|R=1,Y\geq C+\mu_{1}]-𝔼[Y|R=0,Y\geq C+\mu_{0}]\\ & =𝔼[Y|R=1]-𝔼[Y|R=0]=\beta . \end{array}$$

This property will also hold in absence of normality, if the outcome distributions for each group are identical in shape and differ only by a mean shift.

We now consider the TM estimator in the presence of dropout. As the estimator assumes worst value dropout, all missing values ($m_{ij}=1$) are assigned a value smaller than the worst observed outcome:

$$(y_{ij}|m_{ij}=1)\leq \min(y_{ij}|m_{ij}=0).$$

The precise value is unimportant as long as the above inequality holds. The trimmed mean is obtained by taking the average of all observations exceeding the quantile of the trimming proportion, *p*, as in (2), where *p* must now be equal to or exceed the largest observed dropout proportion across the groups:

$$p\geq p_{\min}=\max(\frac{1-n_{m1}}{n_{1}},\frac{1-n_{m0}}{n_{0}}).$$

When the trimming fraction, *p*, is chosen based on the observed dropout proportions (so that $p=p_{\min}$), this is known as adaptive trimming. A more common practice is that of fixed trimming, where *p* is chosen *a priori*, with 50% trimming (p=0.5) a customary choice.^18^

The TM estimator assumes lower value dropout, so that all missing values are contained in the fraction, *p*, that is trimmed away, with no missing values in the trimmed fraction used for estimation (the “strong MNAR assumption”):

(4) $$P(M=1)=\left\{\begin{array}{cc} 0 & \text{if}\;Y>F^{-1}(p)\;\text{(trimmed fraction)}\\ q & \text{if}\;Y\leq F^{-1}(p)\;\text{(trimmed away)} \end{array}\right.,$$

with *q* a probability in the interval [0,1]. When (4) holds, $\hat{\beta_{t}}$ is an unbiased estimator for the TM effect, $\beta_{t}$. In addition, if the group variances are equal (the “location shift assumption”), $\beta_{t}$ is unbiased for β (3). Previously, it has been stressed that the TM estimator estimates an unique estimand—the mean difference of the best X% patients in each treatment group, making it hard to compare with other approaches.^19^, ^20^ In order to identify this TM‐specific estimand, only the strong MNAR assumption is required. Under the additional constraint of the location shift assumption, the TM effect becomes an unbiased estimator of the population mean difference as shown in (3).

We now examine the two sources of bias in the TM estimator. Let $B_{t}$ be the total bias for the TM estimator, representing the deviation of the TM estimate, $\hat{\beta_{t}}$, from the population mean difference, β:

(5) $$B_{t}=𝔼[{\hat{\beta }}_{t}-\beta ]=𝔼[{\hat{\beta }}_{t}-\beta_{t}]+(\beta_{t}-\beta ).$$

The first component results from the violation of the strong MNAR assumption, the second from violation of the location shift assumption. Supplementary Figure S1 (Appendix B) illustrates TM and CCA estimator behavior across four scenarios with varying dropout patterns and equal and unequal treatment arm SDs. Box 1 summarizes the terminology used throughout this article.

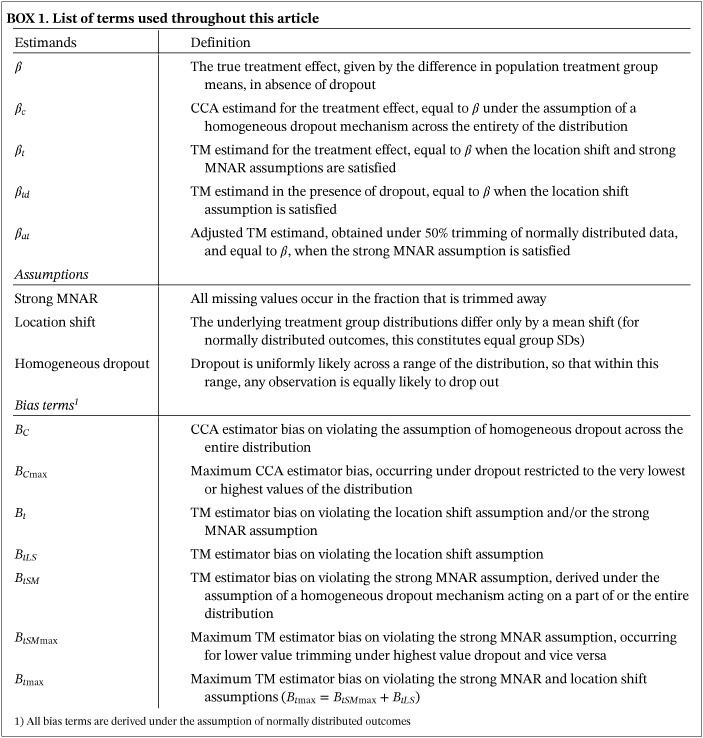

### 2.3 The location shift assumption

We now derive expressions for the bias resulting from the violation of the location shift assumption, $B_{tLS}$, for the case of 50% trimming, with results for a more general trimming fraction, *p*, derived in Appendix C. We assume that the strong MNAR assumption is satisfied and the outcomes are normally distributed for each treatment arm. Then, ${\hat{\beta }}_{t}$ is equal to $\beta_{t}$ and the total bias (5) is given by

$$B_{tLS}=𝔼[{\hat{\beta }}_{t}-\beta ]=\beta_{t}-\beta .$$

We define the 50% trimmed left‐truncated mean (Appendix C):

(6) $$\begin{array}{ll} \mu_{tj} & =\mu_{j}+\sigma_{j}\frac{\phi (\Phi^{-1}(0.5))}{0.5}\\ & =\mu_{j}+\sigma_{j}\sqrt{\frac{2}{\pi }}. \end{array}$$

Then, the 50% trimmed population mean difference is given by

$$\beta_{t}=\left(\mu_{1}+\sigma_{1}\sqrt{\frac{2}{\pi }}\right)-\left(\mu_{0}+\sigma_{0}\sqrt{\frac{2}{\pi }}\right),$$

with the bias, $B_{tLS}$, resulting from unequal SDs:

(7) $$B_{tLS}=(\sigma_{1}-\sigma_{0})\sqrt{\frac{2}{\pi }}.$$

The TM estimator bias, $B_{tLS}$ (7), can alternatively be expressed as a function of the trimmed fraction SDs, rather than of the unobserved full sample SDs (Appendix D):

(8) $$B_{tLS}=(\sigma_{t1}-\sigma_{t0})\sqrt{\frac{2}{\pi -2}}.$$

From both $B_{tLS}$ bias formulae, (7) and (8), it is apparent that the estimator is unbiased given equal treatment group SDs, with the latter notation (8) making it explicit that equality of trimmed fraction SDs suffices. This property underlies the adjustment to the TM estimator outlined in Section 2.5, which relaxes the location shift assumption, allowing for different treatment group SDs.

### 2.4 The strong MNAR assumption

We now derive expressions for the bias resulting from the violation of the strong MNAR assumption, $B_{tSM}$, for 50% lower value trimming and dropout in the comparator group, with results for a given trimming fraction, *p*, and dropout in both groups derived in Appendices D and E, respectively. The strong MNAR assumption assumes that all missing outcomes are restricted to the fraction *p* that is trimmed away, so that all values exceeding the quantile $F^{-1}(0.5)$ are observed:

$$P(M=1)=\left\{\begin{array}{cc} 0\; & \text{if}\;Y>F^{-1}(0.5)\\ q\; & \text{if}\;Y\leq F^{-1}(0.5) \end{array}\right.,$$

with *q* a probability in the interval [0,1].

We assume that the location shift assumption is satisfied and the outcomes are normally distributed in each treatment arm, prior to dropout. Then, $\beta_{t}$ is an unbiased estimator for β and the total bias (5) reduces to

(9) $$B_{tSM}=𝔼[{\hat{\beta }}_{t}-\beta_{t}].$$

We define a new population parameter, $\beta_{td}$, which gives the population TM effect for a scenario with a normally distributed outcome in the treatment group and a comparator group where the outcome is no longer normally distributed due to dropout. Then, ${\hat{\beta }}_{t}$ unbiasedly estimates $\beta_{td}$, the TM effect, and the bias, $B_{tSM}$ (9), can be written as

(10) $$B_{tSM}=\beta_{td}-\beta_{t},$$

and, with $\beta_{t}=\mu_{t1}-\mu_{t0}$ and $\beta_{td}=\mu_{t1}-\mu_{td0}$, simplified to

(11) $$B_{tSM}=\mu_{t0}-\mu_{td0}.$$

Here, $\mu_{t0}$ is the trimmed mean of a comparator group with normally distributed outcomes (6), with for 50% trimming:

$$\mu_{t0}=\mu_{0}+\sigma_{j}\sqrt{\frac{2}{\pi }},$$

and $\mu_{td0}$ the 50% trimmed mean of a comparator group with some non‐normal outcome distribution:

(12) $$\mu_{td0}=𝔼[Y|R=0,Y>F^{-1}(0.5)].$$

We cannot quantify (12) without defining the underlying distribution. If we assume the outcomes are normally distributed prior to dropout, it is sufficient to specify the distribution of the dropout values. In Section 2.4.1 we derive the strong MNAR bias under assumption of a homogeneous dropout mechanism, where, in a given part of the distribution, any observation is equally likely to be selected for dropout. When the homogeneous dropout is confined to the fraction that is trimmed away, the strong MNAR assumption is satisfied. We derive the strong MNAR bias for scenarios where the dropout extends beyond this cutoff. When dropout is homogeneous across the entirety of the distribution, this is equivalent to assuming dropout is MCAR. Section 2.4.2 derives equivalent expressions for bias of the CCA estimator in these scenarios. Section 2.4.3 derives the maximum strong MNAR bias of the TM estimator, which occurs when the missing outcomes are located on the opposite side of the distribution to the one that is trimmed away (eg, if there is 20% dropout and we use the TM estimator under the assumption of 20% lowest value dropout, maximum bias would occur when the 20% highest values drop out).

#### 2.4.1 TM estimator strong MNAR bias under assumption of homogeneous dropout

Consider a truncated normal distribution with lower bound, $\Phi^{-1}(u)$ and upper bound, $\Phi^{-1}(v)$, and let $h_{u,v}$ denote the fraction of this distribution, so that for u=0 and v=1 $h_{0,1}=1$. Let $\mu_{td0}$ be the trimmed mean of a population with normally distributed outcomes, which are no longer normal due to dropout, and let us assume a homogeneous dropout mechanism. Let $h_{0,c}$ denote the fraction affected by homogeneous dropout, with c≤1, and $\Phi^{-1}(c)$ its upper bound, so that each value in $h_{0,c}$ is equally likely to drop out, with a given probability *a*:

$$P(M=1|R=0)=\left\{\begin{array}{cc} a & \text{if}\;Y\leq \Phi^{-1}(c)\\ 0 & \text{if}\;Y>\Phi^{-1}(c) \end{array}\right.,$$

with *a* a probability in the interval [0,1]

Further, let $h_{0,0.5}$ denote the fraction of the distribution that is trimmed away under 50% lower value trimming, $h_{0.5,1}$ the trimmed fraction used for estimation, and $h_{0.5,c}$ the fraction of the distribution for which the trimmed fraction, $h_{0.5,1}$, is affected by dropout (in violation of the strong MNAR assumption).

When the strong MNAR assumption is satisfied, the 50% trimmed mean (6) is given by the top half of the distribution, $\mu_{t}=\mu_{0.5,1}$. When this assumption is violated, dropout is present in the fraction $h_{0.5,1}$, which now contains less than 50% of the outcomes. Consequently, when estimating the 50% trimmed mean, a larger fraction of the distribution, $h_{b,1}$, is taken, with b<0.5, and the trimmed mean given by $\mu_{td}=\mu_{b,1}$. The shift from 0.5 to *b* is a function of the dropout proportion, $p_{d}$, the dropout spread, $h_{0,c}$, the trimming proportion, $h_{0,0.5}=p$, and $h_{0.5,c}=h_{0,c}-p$, with

(13) $$b=h_{0,c}-h_{b,c}=h_{0,c}-\frac{h_{0,c}h_{0,5,c}}{\left(h_{0,c}-p_{d}\right)}.$$

We define the 50% trimmed mean in presence of homogeneous dropout, $\mu_{td}$:

$$\mu_{td0}=\mu_{b,1}=\left(\frac{h_{0.5,c}}{h_{0.5,1}}\mu_{bc}+\frac{h_{c,1}}{h_{0.5,1}}\mu_{c,1}\right),$$

with $\mu_{b,c}$ and $\mu_{c,1}$ the means of truncated normal distributions, with, for example, $\mu_{b,c}$ given by

$$\mu_{b,c}=\mu_{0}-\sigma_{0}\frac{\phi (\Phi^{-1}(c))-\phi (\Phi^{-1}(b))}{c-b}=\mu_{0}-\sigma_{0}Q_{b,c}.$$

The bias of the TM estimator under strong MNAR violation in the comparator group (11) can then be expressed as

(14) $$B_{tSM}=\mu_{0.5,1}-\mu_{b,1}=-\frac{h_{0.5,c}}{h_{0.5,1}}\sigma_{0}(Q_{0.5,c}-Q_{b,c}),$$

$B_{tSM}$ (14) is a function of the size of the trimmed fraction ($h_{0.5,1}$), the fraction affected by dropout ($h_{0.5,c}$), the SD of the affected group ($\sigma_{0}$), and the magnitude of the shift from 0.5 to *b* (13), which is affected by the dropout proportion ($p_{d}$). When the strong MNAR assumption is satisfied, no shift occurs, since b=0.5, so that $Q_{0.5,c}=Q_{b,c}$, and $B_{tSM}$ reduces to 0. The full derivation of (14) is given in Appendix E, with the equivalent for dropout in both treatment groups given in Appendix F.

The bias resulting from the violation of the strong MNAR assumption is determined by the full sample distribution and the dropout mechanism. In (14), we define the bias for normally distributed outcomes and a homogeneous dropout mechanism. When calculating the bias, departures from the strong MNAR assumption are accounted for by increasing the dropout spread. We do this by allowing missing outcomes to be spread over more of the outcome distribution. For example, for 50% lower value trimming, the TM estimator assumes that all dropout occurs in the lower half of the distribution. The analyst may examine sensitivity to this assumption by specifying a dropout spread across the lower 75% of the distribution (c=0.75) and calculating the resulting bias with (14).

This bias is calculated under the assumption that dropout is homogeneous across the specified dropout spread. Under this assumption, the greatest bias occurs when dropout is spread across the entire distribution (*c*=1), which is equivalent to assuming that the outcomes are MCAR. When dropout does depend on a covariate related to the outcome, this will result in violation of the strong MNAR assumption and a non‐homogeneous dropout distribution. Exact quantification of the bias in the latter scenario, however, requires specification of the covariate‐dependent dropout mechanism and the covariate‐outcome relationship and is beyond the scope of this article. Instead, we propose using (14) to calculate an upper bound for the strong MNAR bias. This formula calculates the bias of the TM estimator, which assumes directional dropout, under the assumption that the dropout in truth is random within a given dropout spread.

When there is greater confidence in the strong MNAR assumption holding, the analyst can reflect this by choosing smaller values for *c* in (14), which reduces the assumed dropout spread, resulting in a smaller upper bound for the bias. Supplementary Figure S3 (Appendix G) shows the maximum bias calculated for a range of dropout spreads. In Section 2.4.3, we derive the bias of the TM estimator when outcomes are MNAR contrary to the strong MNAR assumption and located on the opposite side of the distribution to the one being trimmed.

#### 2.4.2 CCA estimator bias under the strong MNAR assumption

Unlike the TM estimator, the CCA estimator will be biased under the strong MNAR assumption. We define this bias, $B_{C}$, for the case of dropout in the comparator group, with results for dropout in both groups derived in Appendix H. As for the TM estimator, we derive the bias under the assumption of a homogeneous dropout mechanism and normally distributed outcomes, with $B_{C}$ given by

(15) $$B_{C}=\beta_{c}-\beta =(\mu_{1}-\mu_{c0})-(\mu_{1}-\mu_{0})=-\mu_{c0}+\mu_{0}=\mu_{0}-\mu_{c0},$$

and

(16) $$\begin{array}{ll} B_{C} & =-\sigma_{0}[\left(h_{0,c}-\frac{\left(h_{0,c}-p_{d}\right)}{\left(1-p_{d}\right)}\right)\frac{\phi (\Phi^{-1}(c)))}{c}\\ & \;+\left((1-h_{0,c})-\frac{\left(1-h_{0,c}\right)}{\left(1-p_{d}\right)}\right)\frac{-\phi (\Phi^{-1}(c))}{1-c}], \end{array}$$

with $h_{0,c}$ the dropout spread, *c* its upper bound, and $p_{d}$ the dropout proportion. We fully derive (16) in Appendix H.

In the absence of dropout ($p_{d}=0$), and for dropout spread homogeneously across the entire outcome distribution ($c=h_{0,c}=1$), $B_{C}$ is 0. The CCA estimate will be maximally biased for dropout restricted to the very lowest or highest values of the distribution, the former resulting in overestimation of the complete case mean, $\mu_{cj}$, the latter in its underestimation. We can write the maximum bias for a given treatment group *j* under high or low value dropout as

(17) $$|B_{C\max}|=\frac{\sigma_{j}}{\left(1-p_{dj}\right)}\phi (\Phi^{-1}(p_{dj})).$$

The full derivation of (17) is given in Appendix I.

The dropout mechanism under which maximum CCA bias is achieved can be defined in terms of a threshold SM. Consider dropout of the $p_{d}$ highest values in the comparator group, so that all observations exceeding the $\left(1-p_{d}\right)$'th quantile of the outcome distribution are unobserved, with an overall selection probability $p_{s}=1-p_{d}$. For normally distributed outcomes, this threshold is given by $t_{H}=\mu_{0}+\sigma_{0}\Phi^{-1}(p_{s})$ so that the probability of selecting a given outcome value is

(18) $$P(M=0|R=0)=\left\{\begin{array}{cc} 0 & \text{if}\;Y\geq t_{H}\\ 1 & \text{if}\;Y<t_{H} \end{array}\right..$$

In the broader context of SMs in general, Copas and Jackson defined a bias limit, which is equivalent to the maximum CCA estimator bias that we derive here (17).^21^

#### 2.4.3 Maximum bias of the TM estimator

We extend Copas and Jackson's bias limit to the TM estimator, and define the maximum possible TM bias, which, for the case of lower value trimming, occurs under highest value dropout. For the comparator group, the maximum bias resulting from dropout is then given by

(19) $$B_{tSM\max}=\frac{-\sigma_{0}}{1-p}[\phi (\Phi^{-1}(p_{s}))-\phi (\Phi^{-1}(p_{s}-(1-p)))+\phi (\Phi^{-1}(1-p))],$$

with the total maximum bias, $B_{t\max}$, accounting for the potential violation of the location shift assumption, given by

(20) $$B_{t\max}=B_{tSM\max}+(\sigma_{1}-\sigma_{0})\frac{\phi (\Phi^{-1}(p))}{1-p}.$$

Full derivations of ((19), (20)) are given in Appendix J, alongside expressions for maximum bias under dropout in the treatment group. Appendix K describes a simple simulation illustrating the maximum CCA and TM estimator biases under dropout assumption violations.

### 2.5 Adjusted estimator

Here, we define an adjusted estimator which relaxes the location shift assumption. Under the assumption of normally distributed outcomes, the 50% trimmed fractions will have half‐normal distributions. The SD of a given fraction can be adjusted by mirroring this half‐normal distribution, and rescaling this now complete, if artificial, normal distribution, using the full sample SD of the other group. The latter SD will either be observed, in the absence of dropout, or can alternatively be extrapolated from the observed fraction SD, using the underlying properties of normality. The adjustment can be performed on either treatment group, but we illustrate the procedure for the comparator group. Let $\mu_{t0}$ be the unadjusted 50% comparator trimmed mean, with

(21) $$\mu_{t0}=\mu_{0}+\sigma_{0}\phi (\Phi^{-1}(0.5))/0.5,$$

and $\mu_{at0}$ the adjusted comparator trimmed mean, with

(22) $$\mu_{at0}=\frac{\mu_{t0}-\mu_{0}}{\sigma_{0}/\sigma_{1}}+\mu_{0}.$$

Substituting (21) for $\mu_{t0}$ in (22) then gives the comparator group trimmed mean under the treatment group SD, $\sigma_{1}$. From the adjusted comparator trimmed mean, $\mu_{at0}$ (22), and the unadjusted treatment trimmed mean, $\mu_{t1}$ (6), we obtain the population adjusted TM estimate, under comparator group rescaling, $\beta_{at0}$, as

$$\beta_{at0}=\mu_{t1}-\mu_{at0}.$$

Equivalently, we can obtain the population adjusted TM estimate, under treatment group rescaling, $\beta_{at1}=\mu_{at1}-\mu_{t0}$, with $\mu_{at1}$ defined analogously to (22). As for the unadjusted estimator, violation of the strong MNAR assumption will bias the estimate, with violation in the comparator group resulting in an overestimation of the treatment effect, and the converse true for the treatment group. We show in simulation (Section 3, Table 1) that violation of the strong MNAR assumption results in greater bias for the adjusted estimators than for the unadjusted estimator, with the greatest bias observed when rescaling the group for which the strong MNAR assumption is most violated, and recommend that the adjustment be applied to the group for which the assumption is most plausible. In Appendix L, expressions are derived for the adjusted estimator bias resulting from strong MNAR assumption violation in either or both treatment groups, under comparator group rescaling and under treatment group rescaling.

**TABLE 1:** Estimated treatment effects ($\hat{\beta }$) for the CCA (${\hat{\beta }}_{c}$), TM (${\hat{\beta }}_{t}$), and adjusted TM (${\hat{\beta }}_{at}$) estimators, with the latter obtained by performing the adjustment on the comparator group (${\hat{\beta }}_{at0}$) and the treatment group (${\hat{\beta }}_{at1}$)

Dropout spread		${\hat{\beta }}_{c}$	$\hat{\beta_{t}}$	${\hat{\beta }}_{at1}$	${\hat{\beta }}_{at0}$	${\hat{B}}_{tLS}^{\;\;a}$	${\hat{B}}_{tSM}^{\;\;\;b}$	${\hat{B}}_{t}^{\;c}$	${\hat{B}}_{at1}^{\;\;d}$	${\hat{B}}_{at0}^{\;\;e}$
(a) $\sigma_{0}=\sigma_{1}=1$										
1) $h_{0,c}=0.2$	$\hat{\beta }$/$\hat{B}$	0.15	0.50	0.50	0.50	0.00	0.00	0.00	0.00	0.00
	$\hat{\text{SD}}$	0.02	0.02	0.03	0.03	0.02	0.02	0.02	0.03	0.03
2) $h_{0,c}=0.5$	$\hat{\beta }$/$\hat{B}$	0.30	0.50	0.50	0.50	0.00	0.00	0.00	0.00	0.00
	$\hat{\text{SD}}$	0.02	0.02	0.03	0.03	0.02	0.02	0.02	0.03	0.03
3) $h_{0,c}=0.75$	$\hat{\beta }$/$\hat{B}$	0.40	0.56	0.64	0.70	0.00	0.06	0.06	0.14	0.20
	$\hat{\text{SD}}$	0.02	0.02	0.03	0.03	0.02	0.02	0.02	0.03	0.03
4) $h_{0,c}=1$	$\hat{\beta }$/$\hat{B}$	0.50	0.69	0.77	0.81	0.00	0.19	0.19	0.27	0.31
	$\hat{\text{SD}}$	0.02	0.02	0.03	0.03	0.02	0.02	0.02	0.03	0.03
(b) $\sigma_{0}=1.5$, $\sigma_{1}=1$										
1) $h_{0,c}=0.2$	$\hat{\beta }$/$\hat{B}$	−0.03	0.10	0.50	0.50	−0.40	0.00	−0.40	0.00	0.00
	$\hat{\text{SD}}$	0.03	0.03	0.03	0.03	0.03	0.03	0.03	0.03	0.03
2) $h_{0,c}=0.5$	$\hat{\beta }$/$\hat{B}$	0.20	0.10	0.50	0.50	−0.40	0.00	−0.40	0.00	0.00
	$\hat{\text{SD}}$	0.03	0.03	0.03	0.03	0.03	0.03	0.03	0.03	0.03
3) $h_{0,c}=0.75$	$\hat{\beta }$/$\hat{B}$	0.34	0.19	0.70	0.82	−0.40	0.09	−0.31	0.20	0.32
	$\hat{\text{SD}}$	0.03	0.03	0.04	0.04	0.03	0.03	0.03	0.04	0.04
4) $h_{0,c}=1$	$\hat{\beta }$/$\hat{B}$	0.50	0.39	0.91	0.96	−0.40	0.29	−0.11	0.41	0.46
	$\hat{\text{SD}}$	0.03	0.03	0.03	0.03	0.03	0.03	0.03	0.03	0.03

*Note*: TM estimator bias when violating the location shift (${\hat{B}}_{tLS}$) and strong MNAR assumptions (${\hat{B}}_{tSM}$) are shown, alongside the total bias (${\hat{B}}_{t}$), and the adjusted TM estimator biases (${\hat{B}}_{at0}$, ${\hat{B}}_{at1}$). Mean and SD estimates for S=1000 simulations are reported. The TM estimands are estimated under 50% trimming of normally distributed outcomes (N=1000), for equal (a) and unequal (b) treatment group SDs, true treatment effect β=0.5, and 20% comparator group dropout. Four scenarios are considered: dropouts restricted to (1) the lowest 20% of the distribution ($h_{ac}=0.2$), (2) the lowest 50%, (3) the lowest 75%, and (4) left unrestricted.

${}^{a}$ ${\hat{B}}_{LS}$ is defined in (7), Section 2.3.

${}^{b}$ ${\hat{B}}_{SM}$ is defined in (14), Section 2.4.

${}^{c}$ ${\hat{B}}_{t}={\hat{\beta }}_{t}-0.5$ and equivalently ${\hat{B}}_{t}={\hat{B}}_{LS}+{\hat{B}}_{SM}$.

${}^{d}$ ${\hat{B}}_{at1}={\hat{\beta }}_{at1}-0.5$, with ${\hat{B}}_{at1}$ further defined in (L14), Appendix L.3.

${}^{e}$ ${\hat{B}}_{at0}={\hat{\beta }}_{at0}-0.5$, with ${\hat{B}}_{at0}$ further defined in (L7), Appendix L.1.

## 3 A SIMULATION STUDY

Consider a clinical trial on n=1000 subjects randomized to either a treatment (j=1) or comparator (j=0) arm, with $n_{1}=n_{0}=500$. We assume the outcomes are normally distributed, with a true treatment effect β=0.5, and 20% dropout in the comparator group. Table 1 gives the mean treatment effect estimates and bias across S=1000 simulations for the CCA estimator and the TM estimator under 50% trimming, for four scenarios. In the first three, dropout is restricted to the lowest 20%, 50%, and 75%, respectively, of the comparator arm outcome distribution, and MCAR in the fourth scenario.

In the first two scenarios, the strong MNAR assumption is satisfied and we observe in Table 1a, for equal treatment SDs ($\sigma_{1}=\sigma_{0}=1$), that the TM estimate, ${\hat{\beta }}_{t}=0.5$, is unbiased, while the CCA estimate, ${\hat{\beta }}_{c}$, is biased towards the null. On violating the strong MNAR assumption, in Scenarios 3 and 4, this CCA estimator bias decreases, with ${\hat{\beta }}_{c}$ being unbiased under random dropout ($h_{ac}=1$). We observe the same pattern for $\sigma_{0}=1.5$ and $\sigma_{1}=1$ (Table 1b), with the CCA bias now larger due to the higher $\sigma_{0}$, which acts multiplicatively on the bias (16). Under unequal SDs, the location shift assumption is violated, and the TM estimates are biased towards the null. For the first two scenarios, the total bias, ${\hat{B}}_{t}$, is equal to ${\hat{B}}_{tLS}$, while in Scenarios 3 and 4, ${\hat{B}}_{t}$ is given by the sum of ${\hat{B}}_{tLS}$ and ${\hat{B}}_{tSM}$, with the latter biasing the TM estimates away from the null. As for the CCA estimator, the increased comparator group SD results in a larger ${\hat{B}}_{tSM}$ bias in Table 1b.

The adjusted TM estimates are given by ${\hat{\beta }}_{at1}$ and ${\hat{\beta }}_{at0}$, for rescaling applied to the treatment and comparator group, respectively. In the first two scenarios, the strong MNAR assumption is satisfied, and both estimators estimate an unbiased treatment effect, $\hat{\beta }=0.5$, under unequal SDs (Table 1b). For Scenarios 3 and 4, however, both are biased away from the null, with bias components, ${\hat{B}}_{at1}$ and ${\hat{B}}_{at0}$, bigger than the unadjusted estimator bias, ${\hat{B}}_{tSM}$. The greatest bias is observed for ${\hat{B}}_{at0}$, a consequence of performing the adjustment on the group for which the strong MNAR assumption is violated.

Examples of bias calculations for the CCA and (un)adjusted TM estimators are given in Appendix M. Appendix N describes an additional simulation, for trimming fractions, *p*, other than 50%, illustrating the bias trade‐off between the $B_{tLS}$ and $B_{tSM}$ bias components. Appendix O provides R code for obtaining adjusted and unadjusted TM estimates for a simulated dataset.

Table 2 shows the results for a set of more complicated simulations, in which an additional continuous covariate, *U*, acts on the outcome (Scenario I). Once again, we impose 20% lower value dropout in the comparator group, but now also allow for covariate‐dependent dropout (Scenario II). Given equal treatment group SDs, the CCA estimate, ${\hat{\beta }}_{c}$, is biased, while the TM estimate, ${\hat{\beta }}_{t}$, is unbiased in Scenarios I and II, for normally and log‐normally distributed outcomes, both for the naive regression of outcome on treatment, and when including *U* (adj) in the regression. Given unequal SDs, the adjusted TM estimate, ${\hat{\beta }}_{at1}$, obtained by rescaling the group with the least dropout, is unbiased for normally distributed outcomes, when dropout is restricted to the trimming fraction (Scenario I). Unlike the regular TM estimator, the adjusted TM estimator does not allow for additional sources of dropout and adjusting for covariates within the regression will introduce a small bias.

**TABLE 2:** Estimated treatment effects for the CCA (${\hat{\beta }}_{c}$), TM (${\hat{\beta }}_{t}$), and adjusted TM (${\hat{\beta }}_{at1}$) estimator, with the latter obtained when performing the adjustment on the treatment group

		Equal SDs				Unequal SDs					
		$\hat{\beta_{c}}$	$\hat{\beta_{t}}$	${\hat{\beta }}_{at1}$		$\hat{\beta_{c}}$	$\hat{\beta_{t}}$	${\hat{\beta }}_{at1}$		$p_{d1}$	$p_{d0}$
Normally distributed outcomes											
I	$\hat{\beta }$	0.30	1.00	1.00		0.13	0.61	1.00		0.00	0.20
	SD	0.13	0.15	0.14		0.15	0.16	0.16		0.00	0.00
I (adj)	$\hat{\beta }$	0.45	1.00	1.00		0.25	0.58	0.97		0.00	0.20
	SD	0.11	0.14	0.14		0.13	0.15	0.15		0.00	0.00
II	$\hat{\beta }$	0.24	1.00	0.83		0.07	0.69	0.92		0.18	0.36
	SD	0.15	0.17	0.19		0.16	0.18	0.19		0.02	0.02
II (adj)	$\hat{\beta }$	0.39	1.00	0.83		0.18	0.66	0.89		0.18	0.36
	SD	0.13	0.16	0.18		0.15	0.17	0.18		0.02	0.02
Log‐normally distributed outcomes											
I	$\hat{\beta }$	0.30	1.00	0.73		0.20	0.63	0.89		0.00	0.20
	SD	0.14	0.19	0.15		0.07	0.10	0.10		0.00	0.00
I (adj)	$\hat{\beta }$	0.46	1.00	0.73		0.33	0.61	0.86		0.00	0.20
	SD	0.13	0.19	0.15		0.03	0.09	0.09		0.00	0.00
II	$\hat{\beta }$	0.35	1.00	0.63		0.14	0.72	0.84		0.18	0.36
	SD	0.15	0.19	0.16		0.09	0.14	0.16		0.02	0.02
II (adj)	$\hat{\beta }$	0.54	1.00	0.63		0.27	0.70	0.82		0.18	0.36
	SD	0.14	0.19	0.16		0.07	0.12	0.14		0.02	0.02

*Note*: Mean and SD estimates for S=1000 simulations are reported. The TM estimands are estimated under 50% trimming of normally and log‐normally distributed outcomes (N=1000), for equal ($\sigma_{0}=\sigma_{1}=2$) and unequal ($\sigma_{0}=2.5$, $\sigma_{1}=2$) treatment group SDs, true treatment effect β=1, and 20% lower value comparator group dropout ($h_{0,c}=0.2$). Two scenarios are considered: (I) the outcome, *Y*, is a function of treatment group and an additional continuous covariate, *U*; (II) the outcome, *Y*, is a function of treatment group and *U*, with *U* also affecting dropout, simulated via a logit missingness mechanism. Dropout proportions are shown for the treatment ($p_{d1}$) and comparator ($p_{d0}$) groups. Unadjusted CCA and TM estimates are obtained, alongside estimates adjusted (adj) for *U* in the regression.

Table 3 considers a scenario in which both outcome and covariate values are missing. In Scenario I, we have 20% lowest value dropout, and treatment dependent missingness in *U*. In Scenario II, we additionally have *U*‐dependent dropout of outcomes. Missingness in *U* results in a reduced sample size for both the CCA estimator ($N_{c}$) and TM estimator ($N_{t}$). In both scenarios, imputing the missing *U* values in the trimmed fraction will give an unbiased treatment effect estimate.

**TABLE 3:** Estimated treatment effects for the CCA (${\hat{\beta }}_{c}$) and TM (${\hat{\beta }}_{t}$) estimators in observed and imputed data

		Observed data						Imputed data				
		$\hat{\beta_{c}}$	SE	$N_{c}$	$\hat{\beta_{t}}$	SE	$N_{t}$	$\hat{\beta_{t}}$	SE	$N_{tI}$	$p_{d1}$	$p_{d0}$
I	Est	0.43	0.13	631	1.00	0.13	358	1.00	0.11	500	0.00	0.20
	SD	0.13	0.00	13.34	0.15	0.01	9.79	0.14	0.01	0.00	0.00	0.00
II	Est	0.39	0.14	510	1.00	0.14	358	1.00	0.12	500	0.18	0.36
	SD	0.15	0.01	14.89	0.17	0.01	10.22	0.15	0.01	0.00	0.02	0.02

*Note*: Mean and SD estimates for S=1000 simulations are reported. The TM estimand is estimated under 50% trimming of normally distributed outcomes (N=1000), for equal ($\sigma_{0}=\sigma_{1}=2$) treatment group SDs, true treatment effect β=1, and 20% lower value comparator group dropout ($h_{0,c}=0.2$). In Scenario I, the outcome, *Y*, is a function of treatment group and an additional continuous covariate, *U*, with approx. 30% missingness in *U* as a function of treatment, simulated via a logit missingness mechanism. Scenario II is equivalent to I, but now *U* also affects dropout, simulated via a logit missingness mechanism. Estimated treatment effects are shown alongside SEs, and the number of cases used in estimation. $N_{c}$ and $N_{t}$ give the number of complete cases (*U* and *Y* not missing) in the observed and trimmed data, respectively. All estimates are adjusted for *U*, and data is imputed after 50% trimming.

## 4 AN APPLICATION TO THE COBALT RANDOMIZED CLINICAL TRIAL

The CoBalT trial^22^, ^23^ was a multicenter trial investigating the effect of CBT as an adjunct to pharmacotherapy vs usual care (UC) in 469 patients aged 18‐75 with treatment‐resistant depression. The primary outcome was the BDI‐II score—a self‐completed measure of depressive symptoms, with higher values indicating greater depression. Long‐term treatment effect was assessed by a repeated measures intention‐to‐treat analysis (N=396), using outcomes at 6 months, 12 months, and 3‐5 years (average 46 months), and by the adjusted mean difference at 46 months. Both estimates were adjusted for BDI‐II at baseline, treatment center, and minimization variables (previously prescribed antidepressants, presence of a counselor at the general practice, and duration of current depressive episode at baseline). The repeated measures analysis showed a beneficial effect of CBT vs UC of −4.7 (95% CI: −6.4,−3.0). At 46 months, the adjusted mean difference was −3.6 (95% CI: −6.6,−0.6) for 136 and 112 patients in the CBT and UC group, respectively, with an average dropout rate of 47.1%.^23^ In this section, we apply the TM estimator as a sensitivity analysis for the treatment effect estimated at 46 months.

### 4.1 Missing data

Just over half of the initially recruited patients were observed at final follow‐up, with greater dropout in the UC group than the CBT group (Table 4). Dropout in the UC group appears to be unrelated to the most recent depression score, whereas in the CBT group, dropout was more common in those with higher recent depression scores (Table 5). The CBT group SDs are higher, most noticeably for patients missing at 46 months, suggesting that the unobserved full sample CBT SD at 46 months may be higher than the UC group SD. In Supplementary Figure S5 (Appendix P), histograms of the BDI‐II score distributions are given across time, for the UC group, the CBT group and their combined total. We observe that the distributions are moderately right‐skewed and non‐normal. No missingness was present in the minimization variables included in the model.

**TABLE 4:** Counts (*N*) and percentages (%) of patients with an observed BDI‐II score at baseline, 6, 12, and 46 months, for the usual care group (UC), the CBT treatment group (CBT), and both groups

	UC		CBT		Total	
	N	%	N	%	N	%
Base	235	100	234	100	469	100
6 months	213	90.6	206	88.0	419	89.3
12 months	198	84.3	197	84.2	395	84.2
46 months	112	47.7	136	58.1	248	52.9

**TABLE 5:** Mean BDI‐II measurements and SDs at baseline, 6, 12, and 46 months for the usual care group (UC) and the CBT treatment group

	Usual care (UC)			CBT		
	Observed^a^	Missing^a^	Difference^b^	Observed^a^	Missing^a^	Difference^b^
Base	30.9 (10.3)	32.7 (11.3)	−1.8 (−4.6,1.0)	30.4 (9.3)	33.6 (11.7)	−3.2 (−5.9,−0.5)
6 months	24.6 (13.1)	24.4 (13.2)	0.2 (−3.3,3.8)	17.4 (13.6)	21.8 (14.9)	−4.5 (−8.5,−0.4)
12 months	21.8 (13.3)	21.5 (12.4)	0.2 (−3.4,3.9)	15.8 (12.9)	19.5 (15.8)	−3.8 (−7.9,0.4)
46 months	23.4 (13.2)			19.2 (13.8)		

*Note*: Patients missing and observed at 46 months are compared for all time points, with mean differences and 95% confidence intervals provided.

${}^{a}$ Given are mean and SD.

${}^{b}$ Given is the mean difference (observed‐missing) with 95% confidence interval.

### 4.2 Analysis details

Using the maximum CCA bias formula (17) from Section 2.4, we would expect the treatment effect to either under‐ or over‐estimate the treatment effect by approximately 10 units of the BDI‐II score. This is a wide bound, obtained without taking into account information available from the data and motivating the use of a more precise sensitivity analysis. To this end, we employed the TM approach and two MI models as a sensitivity analysis for the treatment effect at 46 months, which was estimated using linear regression adjusting for treatment center, BDI‐II at baseline and various minimization variables (previously prescribed antidepressants, presence of a counselor at the general practice, duration of current depressive episode at baseline). At 46 months, there was 41.9% and 52.3% dropout in the CBT and UC groups, respectively. Due to the high dropout proportion, adaptive trimming was employed, with 52.3% of the highest values trimmed away in both groups. Confidence intervals were obtained using a permutation‐based approach.^18^ We considered only the unadjusted TM estimator, as the adjusted estimator (Section 2.5), which adjusts for unequal treatment group SDs, is strongly reliant on normality, whereas for the unadjusted TM estimator it is sufficient that outcomes have the same distribution across treatment groups. The imputation models included auxiliary variables: baseline variables associated with missing BDI‐II at any time of follow‐up, and various measures of depression and anxiety (BDI‐II, PHQ‐7, GAD‐7, SF‐12). Two MI models were considered, with the first using only depression/anxiety information available at baseline (MI‐baseline), the second including intermediate outcome measurements at all follow‐up times. The MI models were fitted using the R software package “mice”.

To interpret the sensitivity analysis results, we used the bias formulae derived in Sections 2.3 and 2.4 to establish CCA and TM estimator bias directions under different dropout scenarios (Section 4.3). Additionally, we calculated the expected TM estimator bias of the CBT treatment effect in the CoBalT data, for three variations of the most plausible dropout scenario. The location shift assumption bias and strong MNAR bias are both functions of the full sample SDs, which remain unobserved. Subject to the specified dropout mechanism, the observed dropout proportions, and under assumption of normally distributed outcomes, these can be inferred from the observed SDs, and used to calculate the relevant bias terms (derivations and R code in Appendix Q). While the TM bias formulae are derived under assumption of normally distributed outcomes and the 46 month BDI‐II scores are skewed, the formulae are sufficient for establishing bias direction and relative changes in bias size across different dropout scenarios.

### 4.3 Plausibility of assumptions for CCA, MI, and TM estimators

**FIGURE 1:**
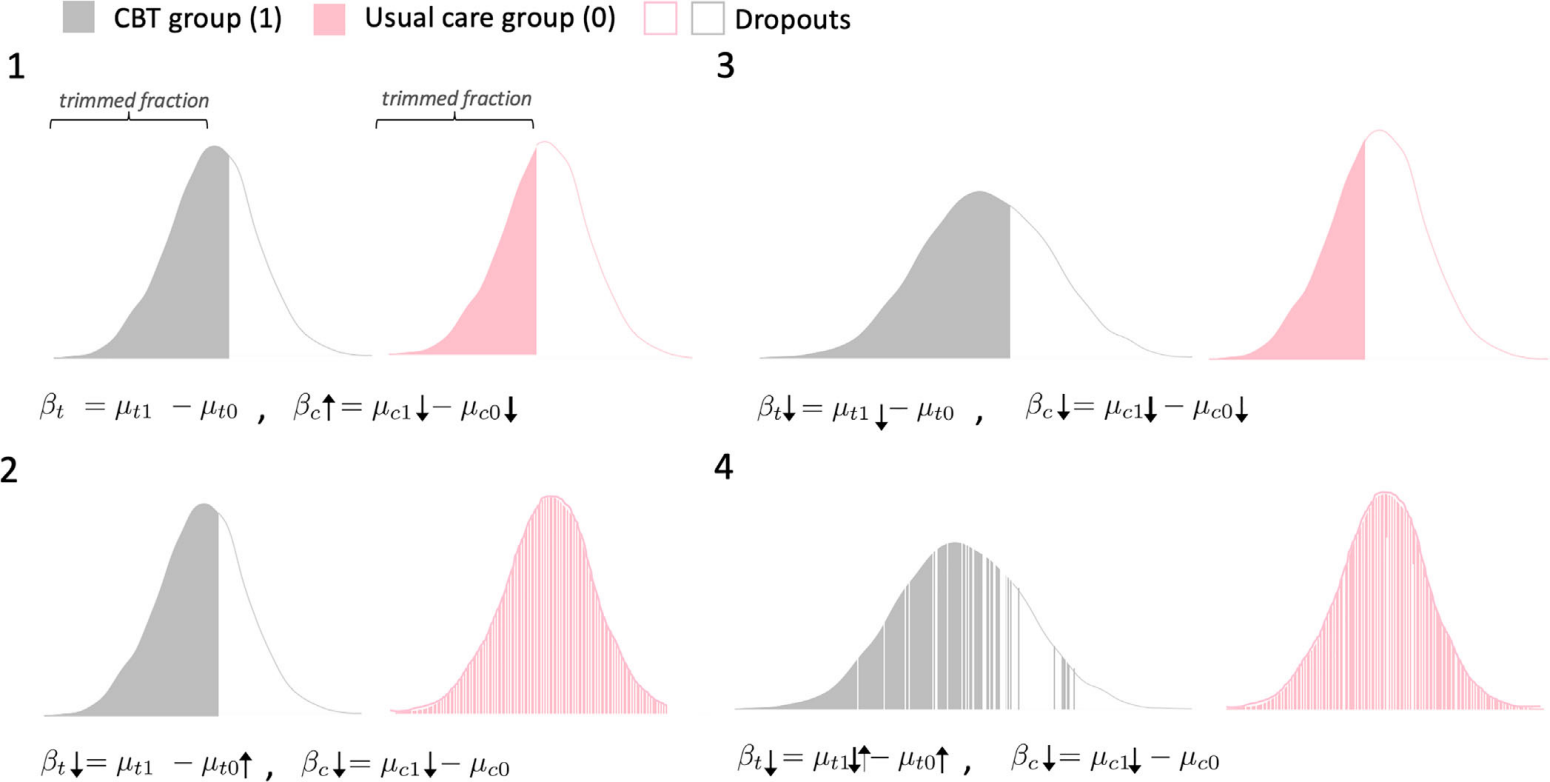
Trimmed means (TM) and complete case analysis (CCA) estimators across four scenarios with varying dropout patterns and treatment arm distributions, for 52.3% dropout in the usual care group (pink), 41.9% dropout in the CBT group (grey) and 52.3% higher value trimming. (1) The location shift assumption and strong MNAR assumption are satisfied, with equal treatment group SDs and strictly higher value dropout. (2) The location shift assumption is satisfied, but the strong MNAR assumption is violated in the usual care group. (3) The location shift assumption is violated, with $\sigma_{1}>\sigma_{0}$, and the strong MNAR assumption is satisfied. (4) The location shift assumption is violated with $\sigma_{1}>\sigma_{0}$, and the strong MNAR assumption is violated in the usual care group and to a lesser degree in the CBT group

The validity of each method rests on the underlying assumptions the estimator makes about data characteristics, which cannot (usually) be tested. We used the bias formulae derived in Sections 2.3 and 2.4 to compare TM and CCA estimator behavior across four plausible dropout scenarios (Figure 1) and to identify the most plausible direction of bias in the CoBalT data with respect to the CBT treatment effect estimated at 46 months. We used this information to interpret the results of the sensitivity analysis in Section 4.4.

The CCA estimate will be unbiased if the outcomes at 46 months are MAR conditional on the minimization variables included in the model, and biased if the outcomes are MNAR. Dropout of more depressed patients in the CBT group will result in underestimation of the CBT complete case mean, with the CCA estimator consequently overestimating the beneficial effect of CBT (Figure 1(2)).

The TM estimator will be unbiased if the location shift assumption and strong MNAR assumption hold (Figure 1(1)). From the CoBalT data, we suspect dropout of patients with more severe depressive symptoms in the CBT group (higher value dropout) and (approximately) homogeneous (MCAR) dropout in the UC group. This will, under higher value trimming, lead to the inclusion of too many high values in the trimmed fraction, and consequently to overestimation of the UC trimmed mean and the treatment effect (Figure 1(2)). Intermediate BDI‐II measurements at earlier times suggest a comparatively higher SD for the CBT group, implying that the location shift assumption may be violated (Figure 1(3)), with the larger spread of values leading to underestimation of the CBT trimmed mean and overestimation of the beneficial effect of CBT. Violation of the location shift assumption, with ${\text{SD}}_{\text{CBT}}>{\text{SD}}_{\text{UC}}$, combined with violation of the strong MNAR assumption in the UC group, will exacerbate the bias, leading to a substantial overestimation of the CBT treatment effect. This is illustrated in Figure 1(4), where the bias is somewhat mitigated by allowing for the possibility that the strong MNAR assumption may also be violated, if to a much lesser extent, in the CBT group.

MI estimates of the CBT treatment effect at 46 months will be unbiased if the outcomes are MAR, conditional on the variables included in the imputation model.^10^, ^12^ Higher‐value dropout in the CBT group, if it cannot be explained by the observed imputation model variables, will result in the imputation of BDI‐II values that are too low in the CBT group, but approximately correct in the UC group, leading to underestimation of the CBT imputed mean. As with the CCA estimator, this will result in a bias away from the null, with the MI estimator overestimating the benefit of CBT treatment. We consider two MI models (Section 4.2), the first including a number of covariates and various depression/anxiety scores measured at baseline, and operating under the assumption that the outcomes at 46 months are missing independently of the true, unobserved outcome, conditional on the outcome measured at baseline and the other covariates. The second MI model additionally includes depression/anxiety measurements at 6 and 12 months, and assumes that the outcomes at 46 months are MAR given all earlier outcome measurements and other covariates. This assumption differs slightly for patients who also have incomplete outcomes at earlier times, with the MAR assumption then implying that the outcomes at 46 months are missing independently of the true outcome, conditional on only the observed previous outcome measures and the other covariates. Under the hypothesis of outcome‐dependent dropout, we would expect the first model to be biased. Under the plausible assumption that a patient's earlier outcomes are correlated with the one measured at 46 months, we would expect this bias to be partly mitigated in the second imputation model, by the inclusion of previous observed depression measures. Unless we are willing to assume, however, that earlier measurements are fully predictive of the final outcome and that dropout at 46 months will not be affected by a patient's BDI‐II score in the period between 12 and 46 months, it is unlikely that the missing outcomes are fully MAR conditional on the imputation variables, resulting in some MNAR bias remaining.

In summary, our bias formulae suggest that under plausible assumptions, TM and CCA will overestimate the treatment effect. Consideration of MI properties suggest that both MI models will do the same, with a lesser bias for the MI model including intermediate depression/anxiety measures.

### 4.4 Results

The CCA treatment effect estimate for CBT of −3.89 (95% CI: −6.95, −0.83) is comparable to the one of the main CoBalT follow‐up analysis^23^ (Table 6). Slightly smaller effects were estimated by the MI models, comparable to the attenuation observed in the MI sensitivity analysis of the CoBalT follow‐up analysis.^23^ The TM estimator, in contrast, estimated a considerably bigger beneficial CBT treatment effect of −8.26 (95% CI: −11.20, −5.32). These results are in line with our expectations regarding the BDI‐II score and dropout characteristics, detailed in Section 4.3.

**TABLE 6:** Treatment effect estimate ($\hat{\beta }$), SE, and 95% confidence interval (95% CI), for the trimmed means (TM) estimator, complete case analysis (CCA), multiple imputation model with baseline outcome measurements (MI baseline) and multiple imputation model with intermediate outcome measurements (MI intermediate)

	$\hat{\beta }$	SE	95% CI
TM	−8.26	1.47	(−11.2, −5.03)
CCA	−3.89	1.53	(−6.92, −0.87)
MI (baseline)	−3.33	1.39	(−6.07, −0.58)
MI (intermediate)	−2.56	1.37	(−5.65, −0.08)

From the CoBalT data, we have reason to suspect higher value dropout in the CBT group and approximately homogeneous dropout in the UC group. Table 7 takes a closer look at the TM estimator bias components calculated under three different assumptions about the dropout mechanism. In Scenario A, we assume completely directional, highest value dropout in the CBT group, with all dropout values (41.9%) restricted to the top of the distribution, and entirely homogeneous dropout in the UC group, with the dropout (53.3%) spread equally across the distribution. In Scenarios B and C, we make less stringent assumptions, with in Scenario B dropout in the top 60% for the CBT group, and in Scenario C dropout in the top 60% of the CBT group and top 80% of the UC group. We obtain a total bias, ${\hat{B}}_{t}$ of −17.53, −11.09, and −7.1 for Scenarios A, B, and C, respectively, and calculate a bias‐adjusted estimate, which can be interpreted as an upper bound for the treatment effect estimate, $\hat{\beta_{t}}$, with the lower bound given by the TM estimate obtained from the data (${\hat{\beta }}_{t}=-8.26)$. For all three scenarios, the CCA and MI estimates (Table 6 ) fall within the $\hat{\beta_{t}}$ bounds, with the bias‐adjusted estimate of Scenario C most comparable, at −1.16. Our sensitivity analysis, comparing CCA, TM, and MI, is consistent with a beneficial CBT treatment effect, but suggests that it may be more modest than indicated by the CCA estimator.

**TABLE 7:** Trimmed mean estimator treatment effect bounds and bias components under different dropout assumptions

	Group	Spread	${\hat{B}}_{LS}$	${\hat{B}}_{SM0}$	${\hat{B}}_{SM1}$	${\hat{B}}_{t}$	${\hat{\beta }}_{tBA}$	TM estimate bounds
A	UC	1	−7.17	−10.36	0	−17.53	9.27	[−8.26, 9.27]
	CBT	0.419						
B	UC	1	−1.14	−10.36	0.41	−11.09	2.83	[−8.26, 2.83]
	CBT	0.6						
C	UC	0.8	−2.01	−5.5	0.41	−7.1	−1.16	[−8.26, −1.16]
	CBT	0.6						

*Note*: (A) CBT group dropout restricted to the top 41.9% of the distribution and homogeneous UC group dropout; (B) CBT group dropout in the top 60% of the distribution and homogenous UC group dropout; (C) CBT group dropout in the top 60% of the distribution and UC group dropout in the top 80%. Shown are the bias components for violation of the location shift assumption (${\hat{B}}_{LS}$), for violation of the strong MNAR assumption in the UC group (${\hat{B}}_{SM0}$), for violation of the strong MNAR assumption in the CBT group (${\hat{B}}_{SM1}$), the total bias (${\hat{B}}_{t}$), the bias adjusted estimate (${\hat{\beta }}_{tBA}$), and the maximum bounds of the TM estimate, ${\hat{\beta }}_{t}$.

## 5 DISCUSSION

In this article we discuss the TM estimator and its use in a sensitivity analysis, when missing data is present in a single continuous outcome variable. Our work extends existing TM literature by deriving formulae that can be used to calculate the bias resulting from violations of the location shift and strong MNAR assumptions, establish bias direction, and also, under certain additional assumptions, calculate a limit for the maximum expected bias. In Section 4.3, we show how these formulae can be used to aid interpretation of sensitivity analyses. In addition to this, we describe an adjustment to the estimator that relaxes the location shift assumption, under the assumption of normally distributed outcomes. While our results apply to normally distributed outcomes, bias formulae can be derived in a similar manner for other distributions. In Section 3, we demonstrate in simulation that the TM estimator remains unbiased when outcomes are non‐normally distributed, when adjusting for other covariates in the model and when performing MI on covariates that are MAR, conditional on the other covariates in the imputation model. Additionally, we show that the TM estimator remains unbiased in the presence of covariate‐dependent dropout in addition to strong MNAR dropout, given that the former is not treatment‐dependent.

Missing data is a common feature in RCTs and may result in biased inference, with any analysis reliant on unverifiable assumptions about the relationship between observed and missing data. To increase confidence in the primary results, their robustness should be assessed by performing sensitivity analyses under a range of plausible alternative assumptions.^2^, ^3^, ^13^, ^14^, ^24^, ^25^ While conducting such analyses in the presence of missing data is recommend practice, only a minority of affected trials report performing any kind of sensitivity analysis. Six review articles describing the handling of missing data in RCTs found that only 22% of the 649 trials reviewed reported some kind of sensitivity analysis.^26^, ^27^, ^28^, ^29^, ^30^, ^31^ The majority retained the missingness assumptions of the original analysis, with only a small subset relaxing the original assumptions, and almost none considering a MNAR mechanism.^26^

Applying sensitivity analyses can be limited by the complexity of the methods, and a lack of transparency about the assumptions underlying each method. Common choices for such analyses are SM or pattern mixture model (PMM) based approaches.^2^ SMs require the specification of the probability of being missing, conditional on the outcome. An example is the weighting approach, described by Carpenter et al, which combines MI with SM for data with missingness in a continuous outcome. Here, a logit SM is specified, given by a linear combination of some function of a fully observed covariate, *X*, and a partially observed outcome, *Y*. Such a model requires specification of the general mechanism (logit), and the contributions of *X* and *Y*, the latter of which is given by a user‐specified parameter that attempts to capture the unknown MNAR missingness mechanism. This approach combines SM with MI by imputing the datasets using standard MI procedure, and then re‐weighting observations with the SM probabilities prior to estimation.^32^, ^33^ PMMs specify the relationship between observed and missing outcomes. An example is the delta‐adjusted PMM, which can be used in conjunction with MI, and assumes, for a continuous outcome, that patients who drop out have a mean outcome that differs by a fixed amount, *delta*, to the ones who remain on study.^15^, ^25^, ^34^ This parameter, *delta*, characterizes the unknown relationship between observed and unobserved data, and is typically informed by expert knowledge (eg, the minimum clinically important difference).^15^, ^16^, ^17^, ^35^ Choosing plausible values and distributions for sensitivity analysis parameters is far from straightforward. At times, a tipping point analysis is employed, which uses a range of *delta* values and examines the point at which the material conclusions change. If this tipping point *delta* is clinically implausible, this encourages greater confidence in the primary results.^25^, ^36^

A simpler alternative to such SM‐ and PMM‐based approaches is to specify some extreme scenarios, and, under these, re‐estimate the main analysis results. An example of such an extreme scenario analysis is the combination approach of best‐worst and worst‐best case sensitivity analyses, in which beneficial outcomes are assigned to dropouts in one group and harmful ones to dropouts in the other group, and vice versa, yielding two estimates under opposing assumptions.^37^, ^38^ The TM estimator, operating under the assumption of strict directional dropout and requiring only the specification of the size of trimming fraction, can be considered part of this family of easily implemented extreme‐case scenario estimators. Such simple bias‐sensitivity analyses will often be sufficient to assess the robustness of inferences to potential bias sources,^24^ but, in the event of ambiguity, can be followed up with a more nuanced and fine‐tuned sensitivity analysis (eg, from the family of SM or PMM models).

While the TM estimator is simpler to implement than SM and PMM, requiring no explicit specification of a missingness model or numerical sensitivity parameters, it is theoretically similar. The TM estimator can be thought of as a threshold SM, with the threshold value informed by the size of the trimming fraction, and with the probability of missing set to zero in the trimmed fraction and left unspecified in the part of the distribution that is trimmed away. A similar parallel can be drawn between TM and the delta‐adjusted PMM, with the *delta* mean difference between observed and missing values implicit in the former and a direct result of the choice of trimming fraction, which is bounded by the largest proportion of missingness observed in either treatment group. For example, given 20% dropout in the comparator group, the minimum trimming fraction is 0.2, and the maximum *delta* is given by the difference between the means of the upper 80% and lower 20% of the outcome value distribution. In delta‐adjusted PMMs two parameters are unknown: the mean difference, *delta*, and the variance of the missing values. Common practice is to set the latter equal to the observed sample variance, and to specify the mean difference, with the choice of *delta* value unrestricted by the model.^39^ The TM estimator, in contrast, requires the specification of only a single bounded parameter and makes no such assumption about the variance of the missing and observed values, instead assuming that the underlying treatment group SDs are equal (the “location shift assumption”).

The TM estimator will give an unbiased estimate of the true treatment effect under the strong MNAR assumption and the location shift assumption. The TM estimator is only unbiased for the particular case of MNAR data where the trimmed fraction remains unaffected by dropout. The TM estimator will generally be biased, even under the null, when outcomes are MAR. An exception to this is when the MAR dropout is non‐differential across treatment groups. Specifically, in an RCT, where baseline covariates can be reasonably expected to be equally distributed across the treatment arms, MAR dropout that is dependent on such a covariate will not result in bias. In practice, missing data will frequently be characterized by more than a single missingness mechanism (eg, missing outcomes may be a mix of MNAR and both treatment‐ and covariate‐dependent MAR). Ocampo et al^19^ sought to resolve this by combining the TM estimator with MI. This approach, however, relies on the assumption that missing values determined by MAR and MNAR can be distinguished using auxiliary information and its success will be heavily affected by the reliability and interpretation of the available information.

As assumptions underlying sensitivity analyses cannot be verified, it is important to know how different underlying data structures affect a given estimator. While previous studies have described the TM assumptions,^18^, ^19^, ^20^ the biases arising from the violation of these strict assumptions have not previously been characterized. By quantifying the bias resulting from violation of these assumptions and establishing bias direction under a range of plausible data scenarios, we show how conclusions can be drawn from a sensitivity analysis, moving beyond a comparison of estimates. For example, in the CoBalT analysis (Section 4.4), the TM estimator, under very strict assumptions, estimated a beneficial effect of CBT treatment of approximately −8. However, when considering more plausible data generating scenarios and relaxing these assumptions, we obtained a bias‐corrected estimate that was much smaller.

In order to identify relevant sensitivity analyses, a framework, such as the one employed for the CoBalT analysis in Section 4.3, should be followed. There, we used bias formulae to interpret preliminary observations from the data (eg, higher value dropout and greater SD in the CBT group), and identify plausible bias directions. Our initial bias results suggested that all estimators would be biased away from the null and overestimate the CBT treatment effect. The logical next step would involve identifying sensitivity analyses that might be biased in the opposite direction for a plausible data scenario. The TM estimator will be particularly useful in cases where it can be expected to be biased in the opposite direction to the CCA analysis. For example, if we had seen evidence of higher value dropout in both groups (ie, previous depression being higher for those with missing outcome data in both UC and CBT groups) and evidence of similar SDs in the two arms, then we would expect the CCA estimator to underestimate the treatment effect given greater dropout in the comparator group, and overestimate the effect given higher dropout in the treatment group, while the TM estimator would remain unbiased for both.

Commonly applied sensitivity analyses employ complex models, for which bias quantification methods are not readily available. In contrast, the TM estimator is a simple approach, for which, as we show, analytic expressions are available for the bias resulting from missingness. In this article, we show how the TM estimator can be used as a sensitivity analysis, by using bias formulae to interpret results in context of information available from the data, as we show in our CoBalT analysis. We recommend performing a TM sensitivity analysis when directional dropout is plausible, using the bias formulae to establish bias direction and interpret estimates.

## CONFLICT OF INTEREST

The authors declare no conflicts of interest.

## Supporting information


**Appendix.** Supporting information

## Data Availability

Data from the CoBalT trial is available on request from the authors
