# Supplementary material for: Sensitivity to missing not at random dropout in clinical trials: Use and interpretation of the trimmed means estimator
Source: Stat Med. 2022 Jan 31;41(8):1462–81. doi: 10.1002/sim.9299 (PMC9303448; doi:10.1002/sim.9299)
Supplement: Supplementary file 1 — Appendix. Supporting information [file SIM-41-1462-s001.pdf]

Appendix

A Example use of the *tmsens* package 3

B An illustration of location shift assumption bias and strong MNAR bias 4

C Bias due to violation of the location shift assumption, for a given trimming fraction, p, as a function of full sample SDs 5

D Bias due to violation of the location shift assumption, as a function of trimmed fraction SDs, for a given trimming fraction, p, and for 50% trimming ($p = 0.5$) 6

E Bias due to violation of the strong MNAR assumption, for dropout in the comparator group, for 50% trimming and for a given trimming fraction, p 7

F Bias due to violation of the strong MNAR assumption, for dropout in both treatment groups 9

G A limited upper bound for bias due to violation of the strong MNAR assumption 10

H CCA bias under homogeneous dropout in one or both intervention groups 11

I CCA bias for dropout restricted to the lowest part of the distribution 13

J Maximum bias of the TM estimator in relation to Copas' and Jackson's bias limit 14

K Simulation illustration: CCA and TM estimator bias for highest value comparator group dropout 16

L Adjusted estimator bias under strong MNAR violation in either or both intervention groups 17

L.1 Adjusted estimator bias on rescaling the comparator group, given strong MNAR violation in the comparator group . . . 17
L.2 Adjusted estimator bias on rescaling the comparator group, given strong MNAR violation in both intervention groups . . . 19
L.3 Adjusted estimator bias on rescaling the intervention group, given strong MNAR violation in the comparator group . . . 19
L.4 Adjusted estimator bias on rescaling the intervention group, given strong MNAR violation in the intervention group and optionally in the comparator group . . . 20
L.5 Obtaining the mirrored full sample SD under strong MNAR assumption violation . . . 21

M Companion to Table 1: Examples of bias calculations 22

M.1 CCA bias . . . 22
M.2 Location shift assumption bias . . . 22
M.3 Strong MNAR assumption bias . . . 23
M.4 Adjusted TM estimator bias . . . 23

N Trade-off of location shift bias and strong MNAR bias under different trimming proportions 25

O R code for obtaining unadjusted and unadjusted TM estimates 26

P Application using the CoBalT RCT: BDI-II score distributions across time and intervention groups 31

Q Inferring the full sample SD from the SD observed under dropout, and calculating bias for the CoBalT application (R code) 31

A Example use of the *tmsens* package

- The package is available from our GitHub repository https://github.com/dea-hazewinkel/tmsens
- Install the package using the remotes package.

```
# install.packages("remotes") # uncomment if remotes not installed
remotes::install_github("dea-hazewinkel/tmsens")
```

- Access the helpfiles for the package.

```
help(package = 'tmsens')
```

- We run the example in the `tm()` function helpfile.
- Simulate data

```
set.seed(123456)
test_dat <- as.data.frame(cbind(c(rep(0, 500), rep(1, 500)),
                          c(sort(rnorm(500, 0, 1)),
                            sort(rnorm(500, 1, 1.5))),
                          rbinom(1000, 2, 0.4), rnorm(1000, 0, 1)))
colnames(test_dat) <- c("TR", "Y", "U", "U2")
head(test_dat)
```

```
##   TR         Y U         U2
## 1  0 -3.765842 1  0.1436833
## 2  0 -2.748868 1 -0.6465657
## 3  0 -2.634001 1 -0.7394814
## 4  0 -2.455813 2 -2.1164507
## 5  0 -2.373603 1 -1.3795743
## 6  0 -2.313400 2  0.8415061
```

- Set the first 200 observations of the outcome variable to missing.

```
test_dat$Y[1:200] <- NA
```

- Fit the trimmed mean estimator.

```
library(tmsens)
tm_obj <- tm(formula= Y ~ TR + U + U2,
             GR = "TR", trF = 0.5, side = "LOW",
             n_perm = 1000, adj_est = TRUE, data = test_dat)
summary(tm_obj)
```

```
##
## Call:
## tm(formula = Y ~ TR + U + U2, GR = "TR", trF = 0.5, side = "LOW",
##     n_perm = 1000, adj_est = TRUE, data = test_dat)
##
##
## Analysis details:
## Treatment (1) group rescaled for adjusted TM estimate
##
## Coefficients:
##        Estimate      Pval 95% CI LO 95% CI HI
## TR     1.354308  0.000000  1.177918     1.531
## U     -0.071352  0.923000 -0.167223     0.025
## U2     0.055791  0.047000 -0.008575     0.120
```

```
## TRadj  0.969812  0.000000  0.840809     1.099
##
##
## Dropout:
## 40%
##
## Sample size after trimming:
## 250
##
## Trimming fraction:
## 50% lower value trimming
```

B An illustration of location shift assumption bias and strong MNAR bias

Supplementary Figure S1 compares the trimmed mean (TM) and complete case analyis (CCA) estimators across single realizations of four scenarios with varying dropout patterns and equal and unequal treatment arm standard deviations (SDs). In Figures S1A and S1B, the TM estimator is an unbiased estimator of the true treatment effect, β, under 50% trimming. The outcome values are normally distributed with underlying population means, μ_0 and μ_1, for comparator and intervention group, respectively, and equal SDs ($\sigma = \sigma_1 = \sigma_0$). Let $\widehat{\mu}_j$ be the mean of the observed outcome in group j, with the CCA estimate given by $\widehat{\beta}_c = \widehat{\mu}_0 - \widehat{\mu}_1$. The TM difference, β_t, is estimated by taking the difference of the observed 50% upper fraction means: $\widehat{\beta}_t = \widehat{\mu}_{t1} - \widehat{\mu}_{t0}$.

In Figure S1A, we observe the full data and in Figure S1B, we have dropout in the comparator group in the lower end of the distribution. For both scenarios, the TM estimator is unbiased, with the true treatment effect ($\beta = 9$) contained within the 95% confidence interval (A: $\widehat{\beta}_t = 9.00$; 95% CI: 8.95,9.04; B: $\widehat{\beta}_t = 9.05$; 95% CI: 8.99,9.09). In contrast, the CCA estimate, calculated from observed means, $\widehat{\mu}_0$ and $\widehat{\mu}_1$, is biased downwards in the presence of worst value dropout ($\widehat{\beta}_c = 8.34$; 95% CI: 8.29,8.40).

Figure S1C illustrates a dropout scenario where the location shift assumption is violated, with the comparator group SD exceeding the intervention group SD. As in S1B, the observed mean, $\widehat{\mu}_0$ overestimates the true mean μ_0, resulting in a CCA estimate biased towards the null ($\widehat{\beta}_c = 7.93$; 95% CI: 7.87,8.00). Now, however, the TM estimate is also biased, as the increased comparator SD inflates the trimmed mean, $\widehat{\mu}_{t0}$, resulting in a TM estimate biased towards the null($\widehat{\beta}_t = 8.16$; 95% CI: 8.09,8.22).

In Figure S1D, the treatment group SDs are once again equal, but now the dropout is no longer restricted to the lower half of the distribution, and consequently the strong missing not at random (MNAR) assumption is violated. Again, both estimates are biased. The CCA estimate underestimates β ($\widehat{\beta}_c = 8.87$; 95% CI: 8.81,8.93), if less so than in Figure S1C, as the dropout is now more evenly spread across the distribution and closer to random dropout. The TM estimate, in contrast, is inflated ($\widehat{\beta}_t = 9.60$; 95% CI: 9.55,9.65).

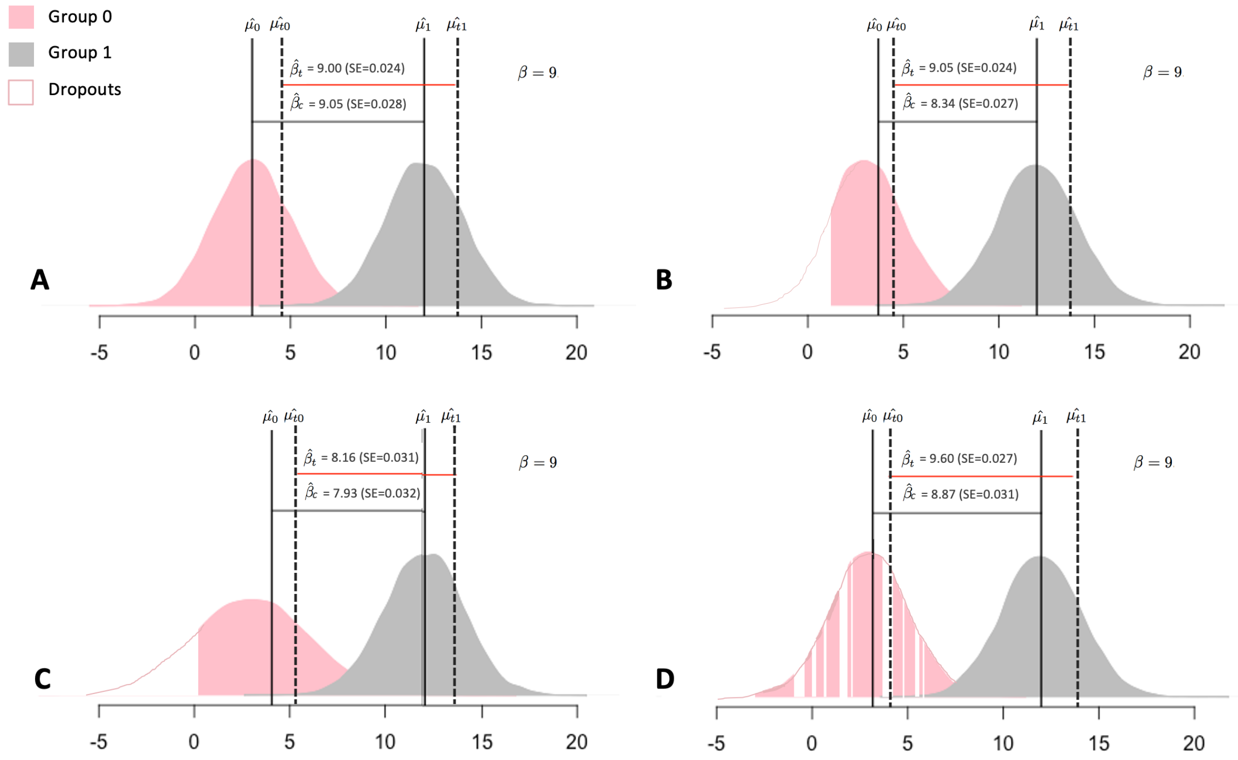

Figure S1: Trimmed means (TM) and complete case analysis (CCA) estimators across four scenarios with varying dropout patterns and treatment arm distributions. A) Equal SDs for the comparator (pink) and intervention group (gray) in the absence of dropout. B) Equal intervention group SDs with 20% lower value dropout in the comparator group. C) Unequal intervention group SDs with a greater comparator group SD and 20% lower value comparator group dropout. D) Equal intervention group SDs with 20% dropout ranging across 90% of the comparator group distribution.

C Bias due to violation of the location shift assumption, for a given trimming fraction, p, as a function of full sample SDs

Let us consider the location shift assumption and its associated bias, B_{tLS}:

$$B_{tLS} = \mathbb{E}[\widehat{\beta}_t - \beta] = \mathbb{E}[\beta_t - \beta]. \tag{C1}$$

For normally distributed outcomes, the trimmed mean, μ_{tj}, for a given intervention group j, can be expressed in terms of a truncated normal distribution. We define the truncated group mean, μ_{tj}, for a distribution with overall mean and SD, μ_j and σ_j, with left- and right-truncation at $\Phi^{-1}(p)$ and $\Phi^{-1}(p_1)$, respectively:

$$\begin{aligned} \mu_{tj} &= \mu_j - \sigma_j \frac{\phi\big(\Phi^{-1}(p_1)\big) - \phi\big(\Phi^{-1}(p)\big)}{\Phi\big(\Phi^{-1}(p_1)\big) - \Phi^{-1}\big(\Phi(p)\big)} \\ &= \mu_j - \sigma_j \frac{\phi\big(\Phi^{-1}(p_1)\big) - \phi\big(\Phi^{-1}(p)\big)}{p_1 - p}. \end{aligned} \tag{C2}$$

Under the assumption of worse value dropout, we define a mean left-truncated at $\Phi^{-1}(p)$ ($p_1 = 1$):

$$\mu_{tj} = \mu_j + \sigma_j \frac{\phi\big(\Phi^{-1}(p)\big)}{1-p}, \tag{C3}$$

Then, the trimmed population mean difference, β_t, for a given trimming fraction, p, is given by

$$\beta_t = \mu_{t1} - \mu_{t0} = \left(\mu_1 + \sigma_1 \frac{\phi\big(\Phi^{-1}(p)\big)}{1-p}\right) - \left(\mu_0 + \sigma_0 \frac{\phi\big(\Phi^{-1}(p)\big)}{1-p}\right), \tag{C4}$$

We define the bias, B_{tLS}, with respect to the population mean difference, $\beta = \mu_1 - \mu_0$, for a given trimming fraction, p:

$$B_{tLS} = (\sigma_1 - \sigma_0) \frac{\phi\big(\Phi^{-1}(p)\big)}{1-p}. \tag{C5}$$

D Bias due to violation of the location shift assumption, as a function of trimmed fraction SDs, for a given trimming fraction, p, and for 50% trimming ($p = 0.5$)

We now define the TM estimator bias, B_{tLS}, in terms of trimmed fraction SDs, for the special case of 50% trimming and for a given trimming fraction, p. Consider the general formula for the variance, σ_{tj}^2, of a truncated normal distribution, with left-truncation at $\Phi^{-1}(p)$ and right-truncation at $\Phi^{-1}(p_1)$:

$$\begin{aligned} \sigma_{tj}^2 &= \sigma_j^2 \Bigg[1 - \frac{\Phi^{-1}(p_1)\phi\big(\Phi^{-1}(p_1)\big) - \Phi^{-1}(p)\phi\big(\Phi^{-1}(p)\big)}{\Phi\big(\Phi^{-1}(p_1)\big) - \Phi\big(\Phi^{-1}(p)\big)} - \\ &\quad \Big(\frac{\phi\big(\Phi^{-1}(p_1)\big) - \phi\big(\Phi^{-1}(p)\big)}{\Phi\big(\Phi^{-1}(p_1)\big) - \Phi\big(\Phi^{-1}(p)\big)}\Big)^2\Bigg] \\ &= \sigma_j^2 \Bigg[1 - \frac{\Phi^{-1}(p_1)\phi\big(\Phi^{-1}(p_1)\big) - \Phi^{-1}(p)\phi\big(\Phi^{-1}(p)\big)}{p_1 - p} - \\ &\quad \Big(\frac{\phi\big(\Phi^{-1}(p_1)\big) - \phi\big(\Phi^{-1}(p)\big)}{p_1 - p}\Big)^2\Bigg]. \end{aligned} \tag{D1}$$

Then, for a left-truncated distribution ($p_1 = 1$), we have

$$\sigma_{tj}^2 = \sigma_j^2 \Bigg[1 - \frac{-\Phi^{-1}(p)\phi\big(\Phi^{-1}(p)\big)}{1-p} - \Big(\frac{-\phi\big(\Phi^{-1}(p)\big)}{1-p}\Big)^2\Bigg], \tag{D2}$$

with for 50% trimming

$$\sigma_{tj}^2 = \sigma_j^2 \Bigg[1 - \Big(\frac{\phi\big(\Phi^{-1}(0.5)\big)}{0.5}\Big)^2\Bigg] = \sigma_j^2 \Bigg[1 - \Big(\sqrt{\frac{2}{\pi}}\Big)^2\Bigg]. \tag{D3}$$

Rearranging (D3) to $\sigma_j^2 = \sigma_{tj}^2/(1-2/\pi)$ and substituting in (C5), with $p = 0.5$, gives the bias, B_{tLS}, as a function of the 50% trimmed fraction SDs:

$$B_{tLS} = (\sigma_{t1} - \sigma_{t0})\sqrt{\frac{2}{\pi - 2}}. \tag{D4}$$

Equivalently, we can obtain a more generalized bias expression for a given trimming fraction, p, by rearranging (D2). Let Z_p denote the quantile of p, with $Z_p = \Phi^{-1}(p)$, giving:

$$\sigma_j^2 = \sigma_{tj}^2 \Big/ \left(1 - \frac{-Z_p\phi(Z_p))}{1-p} - \left(\frac{-\phi(Z_p)}{1-p}\right)^2\right), \tag{D5}$$

Then, by substituting (D5) in (C5), we obtain the location shift assumption bias for a general trimming fraction, p.

$$B_{tLS} = \left[\sqrt{\sigma_{t1}^2 \Big/ \left(1 - \frac{-Z_p\phi(Z_p))}{1-p} - \left(\frac{-\phi(Z_p)}{1-p}\right)^2\right)} - \sqrt{\sigma_{t0}^2 \Big/ \left(1 - \frac{-Z_p\phi(Z_p))}{1-p} - \left(\frac{-\phi(Z_p)}{1-p}\right)^2\right]\right)}\ \right] \frac{\phi\big(\Phi^{-1}(p)\big)}{1-p}. \tag{D6}$$

E Bias due to violation of the strong MNAR assumption, for dropout in the comparator group, for 50% trimming and for a given trimming fraction, p

Consider the bias, B_{tSM}, resulting from the strong MNAR assumption violation, supposing that the location shift assumption is satisfied ($\beta_t = \beta$):

$$B_{tSM} = \mathbb{E}[\widehat{\beta}_t - \beta_t] \tag{E1}$$

In Section 2.4 of the main text, we defined the population parameter, β_{td}, giving the population TM difference for a scenario with normally distributed intervention group outcomes, and comparator group outcomes that are no longer normally distributed due to dropout. When the location shift assumption is satisfied, $\widehat{\beta}_t$ unbiasedly estimates β_{td}, and with $\beta_t = \mu_{t1} - \mu_{t0}$ and $\beta_{td} = \mu_{t1} - \mu_{td0}$, we write the bias (E1) as

$$B_{tSM} = \mathbb{E}[\beta_{td} - \beta_t] = \mu_{t0} - \mu_{td0}. \tag{E2}$$

Consider a normal distribution with lower bound, $\Phi^{-1}(0)$ and upper bound, $\Phi^{-1}(1)$, and let f denote the fraction of this distribution, so that $f_{0,1} = 1$. Then, let $f_{0,0.5}$ denote the fraction of the distribution that is trimmed away under 50% trimming, $f_{0.5,1}$ the trimmed fraction used for estimation, $f_{0,c}$ the fraction affected by dropout, and $f_{0.5,c}$ the fraction of the distribution for which the trimmed fraction, $f_{0.5,1}$, is affected by dropout. We write the trimmed mean in the absence of dropout as the mean of the truncated distribution corresponding to the fraction, $f_{0.5,1}$: $\mu_t = \mu_{0.5,1}$. In the presence of dropout, the fraction, $f_{0.5,1}$, no longer contains a sufficient number of observations to make up the trimmed fraction, and lower values, originally contained in the fraction, $f_{0,0.5}$, are introduced, resulting in a downwards shift of the lower bound, with the trimmed fraction now given by $f_{b,0.5}$, with $b < 0.5$ (see Figure S2). The trimmed mean in presence of dropout is then given by $\mu_{td} = \mu_{b,1}$.

The shift from 0.5 to b is a function of the dropout proportion, p_d, the dropout spread, $f_{0,c}$, the trimming proportion, $f_{0,0.5} = p$, and $f_{0.5,c} = f_{0,c} - p$, with

$$f_{b,c} = \frac{f_{0,c} f_{0.5,c}}{(f_{0,c} - p_d)}, \tag{E3}$$

and

$$b = f_{0,c} - f_{b,c}. \tag{E4}$$

Let us write the TMs as the weighted sum of partial means, and write the trimmed mean in the absence of dropout as $\mu_t = \mu_{0.5,1} = \frac{f_{0.5,c}}{f_{0.5,1}}\mu_{0.5,c} + \frac{f_{c,1}}{f_{0.5,1}}\mu_{c,1}$ and in the presence of dropout as $\mu_{td} = \mu_{b,1} = \frac{f_{0.5,c}}{f_{0.5,1}}\mu_{bc} + \frac{f_{c,1}}{f_{0.5,1}}\mu_{c,1}$. Here, $\mu_{c,1}$ is the mean of a left-truncated normal distribution, and both $\mu_{0.5,1}$ and $\mu_{b,1}$ are the means of bi-truncated normal distributions, with the latter the mean of a truncated normal only under the assumption of homogeneous dropout in the fraction f_{bc}.

We write the bias, B_{tSM} (E2), in terms of weighted partial means:

$$\begin{aligned} B_{tSM} &= \mu_{t0} - \mu_{td0} \\ &= \mu_{0.5,1} - \mu_{b,1} \\ &= \left(\frac{f_{0.5,c}}{f_{0.5,1}}\mu_{0.5,c} + \frac{f_{c,1}}{f_{0.5,1}}\mu_{cd}\right) - \left(\frac{f_{0.5,c}}{f_{0.5,1}}\mu_{bc} + \frac{f_{c,1}}{f_{0.5,1}}\mu_{c,1}\right) \\ &= \frac{f_{0.5,c}}{f_{0.5,1}}\left(\mu_{0.5,c} - \mu_{bc}\right), \end{aligned} \tag{E5}$$

and define μ_{bc} and $\mu_{0.5,c}$ in terms of truncated normal distributions, assuming without loss of generality a zero-centered distribution, with, for example, μ_{bc}, given by

$$\mu_{bc} = \mu_0 - \sigma_0 \frac{\phi\big(\Phi^{-1}(c)\big) - \phi\big(\Phi^{-1}(b)\big)}{c - b} = -\sigma_0 Q_{bc}. \tag{E6}$$

We write the bias (E5) as a function of the size of the trimmed fraction ($f_{0.5,1}$), the fraction affected by dropout ($f_{0.5,c}$) and the SD of the affected group. Then, for dropout in the comparator group:

$$B_{tSM} = -\frac{f_{0.5,c}}{f_{0.5,1}}\sigma_0(Q_{0.5,c} - Q_{bc}). \tag{E7}$$

Equation (E7) gives the bias on violation of the strong MNAR assumption under 50% trimming. For a general trimming fraction, p, we denote the trimmed fraction $f_{p,1}$, and calculate the shift from p to b with

$$b = f_{0,c} - f_{b,c} = f_{0,c} - \frac{f_{0,c} f_{p,c}}{f_{0,c} - p_d}. \tag{E8}$$

The bias is then given by

$$B_{tSM} = -\frac{f_{p,c}}{f_{p,1}}\sigma_0(Q_{p,c} - Q_{bc}). \tag{E9}$$

Figure S2 illustrates the strong MNAR bias mechanism for the case of 50% trimming, with 20% dropout ($p_d = 0.2$) spread across 90% ($f_{0,c} = 0.9$) of the distribution. From (E3), we obtain $f_{b,c} = \frac{0.9\times0.4}{(0.9-0.2)} = 0.51$ and $b = 0.9 - 0.51 = 0.39$. With (E6), we calculate $Q_{0.5,c} = 0.59$ and $Q_{b,c} = 0.34$. Specifying $\sigma_0 = 1$, the bias, B_{tSM} (E7), is then given by $\frac{0.4}{0.5}(0.59 - 0.34) = 0.18$. The bias is positive, and the treatment effect is overestimated, resulting from underestimation of the comparator group mean due to dropout in the trimmed fraction.

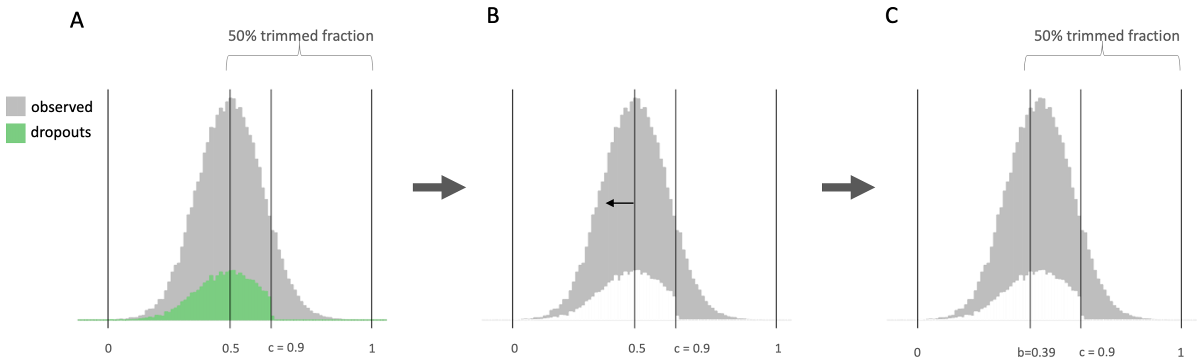

Figure S2: Schematic illustration of the dropout bias mechanism under assumption of homogeneity, for 50% trimming, and 20% dropout ($p_d = 0.2$) spread across 90% of the distribution ($f_{0,c} = 0.9$). A) dropouts (green) and observed patients (gray) for normally distributed outcomes of a given group. The 50% trimmed fraction is given by $f_{0.5,1}$, $f_{0.5,c}$ gives the fraction affected by dropout, $f_{c,1}$ the fraction unaffected by dropout, and $f_{0,0.5}$ the fraction that is trimmed away. B) Under dropout, $f_{0.5,1}$ lacks the observations to make up the 50% trimmed fraction, and the lower boundary, 0.5, shifts downwards in compensation. C) Under dropout, the 50% trimmed fraction is given by $f_{b,1}$. The size of the shift is calculated with (E3) and (E4), giving, for this example, $b = 0.39$.

F Bias due to violation of the strong MNAR assumption, for dropout in both treatment groups

In Section 2.4 of the main text and in Section E of the appendix, we defined the population parameter, β_{td}, which gives the population TM difference for a scenario with normally distributed intervention group outcomes, and comparator group outcomes that are no longer normally distributed due to dropout. Here, we write β_{td} for the population TM difference given strong MNAR assumption violation in both groups, and define the bias as

$$B_{tSM} = \mathbb{E}[\beta_{td} - \beta_t], \tag{F1}$$

with $\beta_t = \mu_{t1} - \mu_{t0}$, and $\beta_{td} = \mu_{td1} - \mu_{td0}$. As dropout is present in both groups, we write:

$$B_{tSM} = (\mu_{td1} - \mu_{t1}) + (\mu_{t0} - \mu_{td0}) = B_{tSM1} + B_{tSM0}, \tag{F2}$$

distinguishing between a intervention group component and comparator group component, with the former given by $B_{tSM1} = \mu_{td1} - \mu_{t1}$ and the latter by $B_{tSM0} = \mu_{t0} - \mu_{td0}$. In Section 2.4 of the main text and Section E of the appendix, we defined the latter in terms of fractions of a truncated normal distribution. We define B_{tSM0} once more below, for a general trimming fraction, p, now using the additional subscript '(0)' to indicate components specific to the comparator group:

$$B_{tSM0} = \frac{f_{p,c(0)}}{f_{p,1}} \left(\mu_{p,c(0)} - \mu_{bc(0)}\right), \tag{F3}$$

with $f_{p,1}$ denoting the trimmed fraction; $f_{p,c(0)}$ the fraction of the comparator distribution for which the trimmed fraction, $f_{p,1}$, is affected by dropout; $\mu_{p,c(0)}$ the mean for the fraction, $f_{p,c(0)}$, in absence of dropout; and $\mu_{bc(0)}$ the mean in the presence of dropout. The fraction bound, $b_{(0)}$, is calculated with (E8) (Section E).

We define the fraction means, $\mu_{p,c(0)}$ and $\mu_{bc(0)}$, in terms of truncated normal distributions, as in (E6) (Section E), and write the comparator group strong MNAR assumption bias component as

$$B_{tSM0} = -\frac{f_{p,c(0)}}{f_{p,1}}\sigma_0(Q_{p,c(0)} - Q_{bc(0)}), \tag{F4}$$

and define the equivalent for the intervention group:

$$B_{tSM1} = -\frac{f_{p,c(1)}}{f_{p,1}}\sigma_1(Q_{p,c(1)} - Q_{bc(1)}). \tag{F5}$$

The total bias, B_{tSM} (F2), is then given by the sum of (F4) and (F5):

$$\begin{aligned} B_{tSM} = B_{tSM0} + B_{tSM1} = -\frac{f_{p,c(0)}}{f_{p,1}}\sigma_0(Q_{p,c(0)} - Q_{bc(0)}) - \\ \frac{f_{p,c(1)}}{f_{p,1}}\sigma_1(Q_{p,c(1)} - Q_{bc(1)}). \end{aligned} \tag{F6}$$

For a given group, j, in the absence of dropout, $b = p$ so that $Q_{bc(j)} = Q_{p,c(j)}$, and the bias reduces to zero. In the event of dropout in the trimmed fraction, $Q_{bc(j)} < Q_{p,c(j)}$, which results in a positive bias component, B_{tSM0} (F4), for the comparator group, and a negative bias component, B_{tSM1} (F5), for the intervention group. For equal dropout proportions across the two groups ($p_{d1} = p_{d0}$) and equal dropout spreads, $Q_{bc(1)} = Q_{bc(0)}$ and $Q_{p,c(1)} = Q_{p,c(0)}$. Then, given equal SDs ($\sigma_1 = \sigma_0$), the bias components are equally sized, but in opposite directions, and the total bias, B_{tSM} (F6), reduces to zero.

G A limited upper bound for bias due to violation of the strong MNAR assumption

Supplementary Figure S3 illustrates the bias that occurs on violation of the strong MNAR assumption in the comparator group, for 20% dropout and under 50% trimming. Figure S3A shows the mean observed bias across $S = 1000$ simulations of sample size $n = 1000$, for a range of dropout spreads and distributions. Figure S3B illustrates the notation used for the fractions, f, of the distribution.

For a given dropout spread, $f_{0,c}$, the bias is plotted against the proportion of dropout present in the part of the trimmed fraction that is affected by dropout, $f_{0.5,c}$. The proportion of dropout in the affected fraction ranges from $p_{d(0.5,c)} = 0$, with no dropout in the affected fraction and by extension the 50% trimmed fraction ($f_{0.5,1}$), to $p_{d(0.5,c)} = 1$, where dropout is spread homogeneously across the entirety of $f_{0,c}$. For example, $p_{d(0.5,c)} = 0.6$ for a dropout spread $f_{0,c} = 0.7$, indicates that only 60% of the dropout remains in the affected fraction, $f_{0.5,c} = 0.2$, when compared to the scenario of entirely homogeneous dropout across $f_{0,c} = 0.7$,

with the missing 40% now allocated to the fraction that is trimmed away ($f_{0,0.5}$). We observe that the TM estimator is increasingly biased the larger the dropout spread, $f_{0,c}$, and the more homogeneous the dropout, with the observed bias (solid line) consistently lower than the calculated limit (dotted line) for all $p_{d(0.5,c)} < 1$ and equal to it for $p_{d(0.5,c)} = 1$.

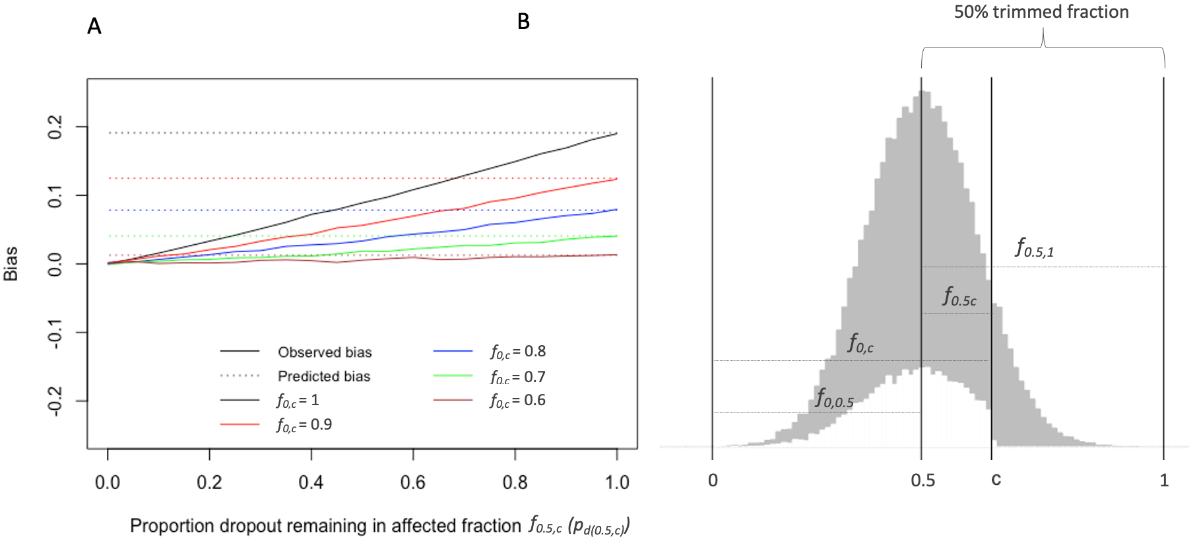

Figure S3: Observed and calculated bias on violating the strong MNAR assumption under 50% trimming. A) Shown is the mean observed bias across $S = 1000$ simulations of sample size $n = 1000$, with equal intervention group SDs of $\sigma_0 = \sigma_1 = 1$ and 20% dropout in the comparator group. The bias is shown for dropout spreads (f_{ac}) of 60 to 100%, across varying dropout distributions, from no dropout in the affected fraction, $f_{0.5,c}$ ($p_{d(0.5,c)} = 0$), to entirely homogeneous dropout across the entirety of $f_{0,c}$ ($p_{d(0.5,c)} = 1$). The bias calculated from (E7, equation 14 in the main text), under the assumption of homogeneity, is shown alongside (dotted line). B) Distributional fractions, f. Observed outcomes are shown in gray, dropouts in white. $f_{0,0.5}$ denotes the fraction that is trimmed away, $f_{0,c}$ the fraction affected by dropout, $f_{0.5,1}$ the trimmed fraction used for estimation, and $f_{0.5,c}$ the part of the trimmed fraction affected by dropout.

H CCA bias under homogeneous dropout in one or both intervention groups

We consider the bias of the CCA estimator, B_C, resulting from violation of the random dropout assumption. Previously, in Section 2.4 of the main text, we defined this bias for dropout restricted to the comparator group. Here, we derive expressions for B_C, for normally distributed outcomes, under dropout in both groups:

$$\begin{aligned} B_C &= \mathbb{E}[\beta_c - \beta] \\ &= (\mu_{C1} - \mu_{C0}) - (\mu_1 - \mu_0) \\ &= (\mu_{C1} - \mu_1) + (\mu_0 - \mu_{C0}) \\ &= B_{C1} + B_{C0}, \end{aligned} \tag{H1}$$

with the intervention group bias component given by B_{C1}, and comparator group bias component given by B_{C0}.

For a given group, j, we define the full sample mean, μ_j (H2), and complete case mean, μ_{Cj}, (H3) as sums of weighted partial means, with

$$\mu_j = f_{0,c}\mu_{0,c} + (1 - f_{0,c})\mu_{c,1}, \tag{H2}$$

and

$$\mu_{Cj} = \frac{(f_{0,c} - p_d)}{(1 - p_d)}\mu_{0,c} + \frac{(1 - f_{0,c})}{(1 - p_d)}\mu_{c,1}, \tag{H3}$$

with $f_{0,c}$ denoting the fraction of the distribution affected by dropout, with $c \leq 1$ (e.g., for dropout in the lower 70% of the distribution, we write $f_{0,c} = 0.7$). With (H2) and (H3), we define the comparator group bias component $B_{C0} = \mu_0 - \mu_{C0}$:

$$B_{C0} = \left(f_{0,c(0)} - \frac{(f_{0,c(0)} - p_{d0})}{(1 - p_{d0})}\right)\mu_{0,c(0)} + \left((1 - f_{0,c(0)}) - \frac{(1 - f_{0,c(0)})}{(1 - p_{d0})}\right)\mu_{c,1(0)}, \tag{H4}$$

with $f_{0,c(0)}$ the dropout spread, p_{d0} the dropout proportion, $\mu_{0,c(0)}$ the mean of the distribution fraction affected by dropout and $\mu_{c,1(0)}$ the mean of unaffected fraction. We write the partial means in terms of a truncated normal distribution, assuming without loss of generality a zero-centered distribution, e.g., for $\mu_{c,1}$ we write

$$\mu_{c,1} = -\sigma\frac{\phi\big(\Phi^{-1}(1)\big) - \phi\big(\Phi^{-1}(c)\big)}{1 - c}. \tag{H5}$$

With (H4) and (H5), we define the bias component for the comparator fully:

$$\begin{aligned} B_{C0} = &-\sigma_0\Bigg[\left(f_{0,c(0)} - \frac{(f_{0,c(0)} - p_{d0})}{(1 - p_{d0})}\right)\frac{\phi\big(\Phi^{-1}(c_0)\big))}{c_0} + \\ &\left((1 - f_{0,c(0)}) - \frac{(1 - f_{0,c(0)})}{(1 - p_{d0})}\right)\frac{-\phi\big(\Phi^{-1}(c_0)\big)}{1 - c_0}\Bigg]. \end{aligned} \tag{H6}$$

In the same manner, we derive the bias component for the intervention group, $B_{C1} = \mu_{C1} - \mu_1$:

$$\begin{aligned} B_{C1} = &-\sigma_1\Bigg[\left(\frac{(f_{0,c(1)} - p_{d1})}{(1 - p_{d1})} - f_{0,c(1)}\right)\frac{\phi\big(\Phi^{-1}(c_1)\big))}{c_1} + \\ &\left(\frac{(1 - f_{0,c(1)})}{(1 - p_{d1})} - (1 - f_{0,c(1)})\right)\frac{-\phi\big(\Phi^{-1}(c_1)\big)}{1 - c_1}\Bigg]. \end{aligned} \tag{H7}$$

Given non-random dropout ($p_{dj} > 0$, $f_{ac(j)} < 1$), B_{C0} will be negative, indicating underestimation of the treatment effect as a consequence of overestimation of complete case comparator mean, μ_{C0}, with the converse true for the intervention group bias component, B_{C1}. The total bias is then given by the sum of (H6) and (H7), where, under equal dropout proportions ($p_{d1} = p_{d0}$), equal dropout spreads ($f_{ac(1)} = f_{ac(0)}$) and equal SDs ($\sigma_1 = \sigma_0$), the bias components will cancel each other out. In the absence of dropout in either group ($p_{d1} = p_{d0} = 0$) or homogeneous dropout in both groups ($f_{ac(1)} = f_{ac(0)} = 1$), both bias components will reduce to zero.

I CCA bias for dropout restricted to the lowest part of the distribution

Here, we derive the CCA bias when dropout is restricted to the lowest part of the distribution, for normally distributed outcomes, for the case of dropout in the comparator group. We define B_{C0}, as in (H6):

$$
\begin{aligned}
B_{C0} = -\sigma_0 \Bigg[\left(f_{0,c(0)} - \frac{(f_{0,c(0)} - p_{d0})}{(1 - p_{d0})} \right) \frac{\phi\big(\Phi^{-1}(c0)\big)}{c0} + \\
\left((1 - f_{0,c(0)}) - \frac{(1 - f_{0,c(0)})}{(1 - p_{d0})} \right) \frac{-\phi\big(\Phi^{-1}(c0)\big)}{1 - c0} \Bigg].
\end{aligned}
\tag{I1}
$$

with $f_{0,c(0)}$ the dropout spread, p_{d0} the dropout proportion, and c_0 the upper bound of $f_{0,c(0)}$, with $c_0 \leq 1$ for a distribution bounded by $\Phi^{-1}(0)$ and $\Phi^{-1}(1)$.

For the special case of dropout restricted to the very lower end of the distribution, $f_{0,c(0)} = p_{d0} = c_0$, and (I1) reduces to

$$
\begin{aligned}
B_{C0} &= -\sigma_0 \left[p_{d0} \frac{\phi\big(\Phi^{-1}(p_{d0})\big)}{p_{d0}} - p_{d0} \frac{-\phi\big(\Phi^{-1}(p_{d0})\big)}{1 - p_{d0}} \right] \\
&= -\sigma_0 \frac{\phi\big(\Phi^{-1}(p_{d0})\big)}{1 - p_{d0}}.
\end{aligned}
\tag{I2}
$$

Lower value dropout leads to overestimation of the complete case mean, μ_{C0}, and underestimation of the treatment effect, reflected in a negative CCA bias component, B_{C0}. The same bias component can be calculated for dropout in the intervention group, by substituting (H7) for (I1) and solving from there, giving:

$$
B_{c1} = \sigma_1 \frac{\phi\big(\Phi^{-1}(p_{d1})\big)}{1 - p_{d1}}. \tag{I3}
$$

For lower value dropout in the intervention group, the bias, B_{c1}, will be positive, as the intervention group complete case mean, μ_{C1}, and, by extension, also the treatment effect are overestimated.

We can apply the same process to dropout at the upper end of the distribution. The resulting bias, owing to the symmetry of the normal distribution, is equal in size and opposite to the bias calculated under the assumption of lowest value dropout (e.g., if for lowest value dropout, $B_{C1} = x$, then for highest value dropout, $B_{C1} = -x$). For a given intervention group, and dropout restricted to either the very lowest or highest values, the group-specific bias component, B_{Cj}, is the maximum bias and given by

$$
\mid B_{Cj} \mid = \frac{\sigma_{0j}}{(1 - p_{dj})} \phi\big(\Phi^{-1}(p_{dj})\big). \tag{I4}
$$

J Maximum bias of the TM estimator in relation to Copas' and Jackson's bias limit

Copas & Jackson (2004) defined a maximum bias for selection models, and proved that this bias limit is achieved when the selection model takes the form of a threshold function, with some overall selection probability p_s, and the probability of selecting a given outcome value going from 0 to 1 at a threshold, t:

$$P(M=0) = \begin{cases} 1 & \text{if } y \geq t \\ 0 & \text{if } y < t. \end{cases} \tag{J1}$$

They define the maximum bias, $B_{\max}$, as a function of the full sample SD, σ and the overall selection probability, p_s:

$$|B_{\max}| = \frac{\sigma}{p_s}\phi\big(\Phi^{-1}(p_s)\big). \tag{J2}$$

The maximum bias for a given group under the CCA estimator (I4) is obtained when there is either strict lowest value dropout or strict highest value dropout (Section I), and is equivalent to Copas and Jackson's maximum bias. Here, we extend this formula and derive expressions for the maximum bias of the TM estimator.

We consider the TM estimator in context of a threshold selection model. In order for the strong MNAR assumption to be satisfied, it is sufficient that all dropouts are contained in the fraction, p, that is trimmed away, or, equivalently, that the trimmed fraction used for estimation $(1-p)$ is completely observed. When trimming under assumption of lower value dropout, all dropout values $(M=1)$ should be lower than the distribution quantile corresponding to p:

$$P(M=1|R=j) = \begin{cases} [0,1] & \text{if } Y < \mu + \sigma\Phi^{-1}(p) \\ 0 & \text{if } Y \geq \mu + \sigma\Phi^{-1}(p). \end{cases} \tag{J3}$$

The bias resulting from the violation of (J3) is a function of the trimmed fraction, $1-p$, and the overall selection probability, p_s, which is equal to $1-p_d$, with p_d the dropout proportion. We define the maximum possible TM estimator bias, which, for lower value trimming, occurs under highest value dropout. Then, for p_d highest value dropout, all observations exceeding the quantile corresponding to $1-p_d$ are unobserved $(M=1)$:

$$P(M=1|R=j) = \begin{cases} 1 & \text{if } Y \geq \mu + \sigma\Phi^{-1}(1-p_d) \\ 0 & \text{if } Y < \mu + \sigma\Phi^{-1}(1-p_d). \end{cases} \tag{J4}$$

The TM estimator bias is then given by the difference between the mean, μ_{td}, obtained under the selection model of (J4), but the assumption of (J3), and the mean, μ_t, obtained under the selection model of (J3). Then, for p_d highest value dropout, and lower value trimming of fraction, p, μ_{td} is calculated over the $1-p$ trimmed fraction, which has lower bound $(1-p_d)-(1-p) = p_s-(1-p)$ and upper bound $(1-p_d) = p_s$. We define the maximum bias, $B_{tSM0\max}$, under highest value dropout in the comparator group and lower value p trimming, denoting the components specific to the comparator group with a subscript '0':

$$B_{tSM0\max} = \mu_{t0} - \mu_{td0}, \tag{J5}$$

with

$$\mu_{t0} = \mu_0 + \sigma_0 \frac{\phi\big(\Phi^{-1}(p)\big)}{1-p}, \tag{J6}$$

and

$$\mu_{td0} = \mu_0 - \sigma_0 \frac{\phi\big(\Phi^{-1}(p_{s0})\big) - \phi\big(\Phi^{-1}(p_{s0} - (1-p))\big)}{1-p}, \tag{J7}$$

with $p_{s0} = 1 - p_{d0}$.

The maximum bias is then given by

$$B_{tSM0\max} = \frac{\sigma_0}{1-p}\Big[\phi\big(\Phi^{-1}(p)\big) + \phi\big(\Phi^{-1}(p_{s0})\big) - \phi\big(\Phi^{-1}(p_{s0} - (1-p))\big)\Big]. \tag{J8}$$

Equation (J8) extends Copas' and Jackson's original formula (J2) to the TM estimator, giving the bias on maximal violation of the strong MNAR assumption. To account for potential violations of the location shift assumption bias, we add the previously defined B_{tLS} bias component (C5):

$$\begin{aligned} B_{t0\max} = & \frac{\sigma_0}{1-p}\Big[\phi\big(\Phi^{-1}(p)\big) + \phi\big(\Phi^{-1}(p_{s0})\big) - \phi\big(\Phi^{-1}(p_{s0} - (1-p))\big)\Big] + \\ & \Big(\sigma_1 - \sigma_0\Big)\frac{\phi\big(\Phi^{-1}(p)\big)}{1-p}. \end{aligned} \tag{J9}$$

Equivalently, we derive the maximum bias for dropout in the intervention group, with $B_{tSM1\max}$ given by

$$B_{tSM1\max} = \mu_{td1} - \mu_{t1}. \tag{J10}$$

Then, with μ_{t1} and μ_{td1} defined analogously to μ_{t0} (J6) and μ_{td0} (J7),

$$B_{tSM1\max} = \frac{\sigma_1}{1-p}\Big[-\phi\big(\Phi^{-1}(p)\big) - \phi\big(\Phi^{-1}(p_{s1})\big) + \phi\big(\Phi^{-1}(p_{s1} - (1-p))\big)\Big], \tag{J11}$$

and

$$\begin{aligned} B_{tSM1\max} = & \frac{\sigma_1}{1-p}\Big[-\phi\big(\Phi^{-1}(p)\big) - \phi\big(\Phi^{-1}(p_{s1})\big) + \phi\big(\Phi^{-1}(p_{s1} - (1-p))\big)\Big] + \\ & \Big(\sigma_1 - \sigma_0\Big)\frac{\phi\big(\Phi^{-1}(p)\big)}{1-p}. \end{aligned} \tag{J12}$$

References

Copas J, Jackson D. A bound for publication bias based on the fraction of unpublished studies. *Biometrics.* 2004;60(1):146-153.

K Simulation illustration: CCA and TM estimator bias for highest value comparator group dropout

Consider a clinical trial on $n = 1000$ subjects, randomized to an intervention ($j = 1$) and comparator ($j = 0$) arm, with $n_1 = n_0 = 500$, normally distributed outcomes, y, a true treatment effect $\beta = 0.5$ and 20% strict highest value dropout in the comparator group. Supplementary Table S1 gives a simulated example ($S = 1000$) for three different comparator group SDs (σ_0) with the intervention group SD (σ_1) held constant. Mean treatment effect estimates are obtained for the CCA ($\widehat{\beta}_c$) and TM estimators ($\widehat{\beta}_t$), for the latter under 50% lower value trimming.

Table S1: Treatment effects for normally distributed outcomes with treatment and comparator group SDs $\sigma_1 = 1$ and $\sigma_0 = 0.5, 1, 1.5$, true treatment effect $\beta = 0.5$, for 20% highest value comparator group dropout, and 50% lower value trimming. Shown are the mean CCA ($\widehat{\beta}_c$) and TM ($\widehat{\beta}_t$) estimates across $S = 1000$ simulations ($N = 1000$). Also shown are the bias components: the CCA estimator bias, $\widehat{B}_C$; the TM estimator location shift assumption bias, $\widehat{B}_{tLS}$; the TM estimator strong MNAR bias, $\widehat{B}_{tSM}$; the total TM estimator bias, $\widehat{B}_t$.

σ_0	$\widehat{\beta}$	$\widehat{\beta}_c$	$\widehat{\beta}_t$	$\widehat{B}_c$	$\widehat{B}_{tLS}$	$\widehat{B}_{tSM}$	$\widehat{B}_t$
0.5	0.50	0.68	1.23	0.18	0.40	0.33	0.73
1	0.50	0.85	1.16	0.35	0.00	0.66	0.66
1.5	0.50	1.03	1.10	0.53	-0.40	0.99	0.59

For the CCA estimator, higher value dropout in the comparator group will lead to underestimation of the comparator complete case mean and overestimation of the treatment effect. In Supplementary Table S1, we see that $\widehat{\beta}_c > \widehat{\beta}$ for all comparator group SDs, with the greatest bias for $\sigma_0 = 1.5 > \sigma_1$. Consider the maximum CCA bias, previously defined in (I3). For highest value dropout in the comparator group, we can then write

$$B_{C0\max} = \frac{\sigma_0}{(1 - p_d)} \phi\big(\Phi^{-1}(p_d)\big). \tag{K1}$$

Filling in (K1), with $\sigma_0 = 1$ and $p_d = 0.2$, gives

$$B_{C0\max} = \frac{1}{(1 - 0.2)} \phi\big(\Phi^{-1}(0.2)\big) = 0.35,$$

which is equal the mean CCA bias for $\sigma_0 = 1$, $\widehat{B}_C = 0.35$, observed in the simulation.

For the TM estimator, higher value dropout in the comparator group, given lower value trimming, will also lead to underestimation of the comparator trimmed mean and overestimation of the treatment effect. This effect can either be exacerbated or mitigated by unequal intervention group SDs, with $\sigma_0 < \sigma_1$ giving a positive location shift assumption bias component, B_{tLS}, and $\sigma_0 > \sigma_1$ giving a negative component, B_{tLS}.

In Supplementary Table S1, we observe positive bias components, $\widehat{B}_{tSM}$, resulting from strong MNAR assumption violation, with the greatest bias for the largest σ_0. We observe a positive location shift assumption bias component, $\widehat{B}_{tLS} = 0.40$ for $\sigma_0 = 0.5 < \sigma_1$, and a negative component, $\widehat{B}_{tLS} = -0.40$ for $\sigma_0 = 1.5 > \sigma_1$, with the total observed TM estimator bias given by $\widehat{B}_t$. Consider the maximum TM estimator bias on violation of the strong MNAR assumption, previously defined in (J8):

$$B_{tSM0\max} = \frac{\sigma_0}{1 - p} \Big[\phi\big(\Phi^{-1}(p_s)\big) - \phi\big(\Phi^{-1}(p_s - (1 - p))\big) + \phi\big(\Phi^{-1}(1 - p)\big) \Big]. \tag{K2}$$

Filling in $p = 0.5$, $p_s = 1 - p_d = 0.8$ and $\sigma_0 = 1$, gives

$$B_{tSM0\max} = \frac{1}{0.5}\Big[\phi\big(\Phi^{-1}(0.8)\big) - \phi\big(\Phi^{-1}(0.3)\big) + \phi\big(\Phi^{-1}(0.5)\big)\Big] = 0.66,$$

which corresponds to the strong MNAR bias component of the TM estimator, $\widehat{B}_{tSM}$, observed for $\sigma_0 = 1$ in Supplementary Table S1.

L Adjusted estimator bias under strong MNAR violation in either or both intervention groups

Here, we define the adjusted estimator bias for the following four scenarios: **L.1**) the bias on rescaling the comparator group, given strong MNAR violation in the comparator group; **L.2**) the bias on rescaling the comparator group, given strong MNAR violation in both the comparator and intervention groups; **L.3**) the bias on rescaling the intervention group, given strong MNAR violation in the comparator group; **L.4**) the bias on rescaling the intervention group, given strong MNAR violation in the intervention group and optionally the comparator group. In Section **L.5**, formulae are derived for the mirrored full sample variance, σ^2_{mj}, for a given group j, in the absence and presence of dropout in the mirrored fraction.

L.1 Adjusted estimator bias on rescaling the comparator group, given strong MNAR violation in the comparator group

In Section 2.5 of the main text, we defined the adjusted TM estimator for comparator group rescaling, and showed that under the strong MNAR assumption, this adjustment gives an unbiased treatment effect estimate when the location shift assumption is violated (equation 22 in the main text). This adjustment is performed for 50% trimming. Under the assumption of normally distributed outcomes, the 50% trimmed fraction will be distributed half-normally, and the SD of a given group can be adjusted by mirroring the fraction and rescaling the resulting distribution, using the full sample SD of the other group. The population adjusted TM estimate, on rescaling the comparator group, is given by

$$\beta_{at0} = \mu_{t1} - \mu_{at0}, \tag{L1}$$

with μ_{t1} the unadjusted 50% intervention group TM, and μ_{at0} the adjusted 50% comparator group TM:

$$\mu_{at0} = \frac{\mu_{t0} - \mu_0}{\sigma_0/\sigma_1} + \mu_0, \tag{L2}$$

with μ_{t0} the unadjusted 50% comparator TM:

$$\mu_{t0} = \mu_0 + \sigma_0\phi\big(\Phi^{-1}(0.5)\big)/0.5. \tag{L3}$$

We now consider this adjustment under strong MNAR assumption violation. The full sample SD (σ_0) and mean (μ_0) in (L2) remain unobserved and are inferred in the process of mirroring. We denote the SD and mean of the mirrored distribution σ_{m0} and μ_{m0}, and write μ_{t0} as μ_{td0}, which is the 50% comparator trimmed mean under strong MNAR assumption violation. The adjusted trimmed mean under strong MNAR assumption violation can then be written as

$$\mu_{atd0} = \frac{\mu_{td0} - \mu_{m0}}{\sigma_{m0}/\sigma_1} + \mu_{m0}, \tag{L4}$$

with σ_1 being the full sample SD from the intervention group, which is either observed in absence of intervention group dropout, or extrapolated from the 50% trimmed fraction. The adjusted TM estimate is given by

$$\beta_{at0d} = \mu_{t1} - \mu_{atd0}.$$

We define the adjusted estimator bias, B_{at0}, for dropout in the comparator group, and under comparator group rescaling:

$$\begin{aligned} B_{at0} &= \beta_{at0d} - \beta_{at0} \\ &= (\mu_{t1} - \mu_{atd0}) - (\mu_{t1} - \mu_{at0}) \\ &= \mu_{at0} - \mu_{atd0}. \end{aligned} \tag{L5}$$

Substituting (L2) and (L4) in (L5), we obtain:

$$\begin{aligned} B_{at0} &= \mu_{at0} - \mu_{atd0} \\ &= \Big[\frac{\mu_{t0} - \mu_0}{\sigma_0/\sigma_1} + \mu_0\Big] - \Big[\frac{\mu_{td0} - \mu_{m0}}{\sigma_{m0}/\sigma_1} + \mu_{m0}\Big]. \end{aligned} \tag{L6}$$

From (L6) we note that μ_{at0} and μ_{atd0} will be identical when $\mu_{td0} = \mu_{t0}$, $\sigma_{m0} = \sigma_0$, and $\mu_{m0} = \mu_0$. These equalities are subject to the strong MNAR assumption and the assumption of distributional normality holding. On violation of the former, μ_{td0} is underestimated, and σ_{m0} overestimated, resulting in the underestimation of μ_{atd0}, and a positive bias, B_{at0}.

Let us write the mean of a given distributional fraction, a to b, as $-\sigma_j Q_{ab}$, with the Q-term the mean of the standard normal for the relevant fraction, expressed in terms of the pdf and quantile functions ($Q_{ab} = \big(\phi\big(\Phi^{-1}(b)\big) - \phi\big(\Phi^{-1}(a)\big)\big)/(b-a)$). From (L2) (equation 22 in the main text), we have μ_{at0}, which is the comparator 50% trimmed mean under the treatment SD, σ_1. Assuming, without loss of generality, a zero-centered distribution, we write $\mu_{at0} = -\sigma_1 Q_{0.5,1}$, and $\mu_{td0} = -\sigma_0(f_1 Q_{bc} + f_2 Q_{c,1})$, where μ_{td0} is the comparator 50% trimmed mean under violation for the strong MNAR assumption, written as the sum of weighted fraction means, for fractions $f_1 = f_{0.5,c}/f_{0.5,1}$ and $f_2 = f_{c,1}/f_{0.5,1}$, and with b obtained from equations (E3) and (E4) in Section E. For a zero-centered distribution, no dropout and strict normality, the comparator mean of the mirrored 50% trimmed fraction, μ_{0m}, is given by $\mu_{0m} = \sigma_0 Z_b = \sigma_0 Z_{0.5} = 0$, with $Z_{0.5}$ the 0.5 quantile of the standard normal distribution. Under strong MNAR assumption violation, $b < 0.5$ and $Z_b < 0$.

We define B_{at0} (L6), fully as

$$B_{at0} = -\sigma_1 Q_{0.5,1} - \Big(- \frac{\sigma_1\sigma_0}{\sigma_{m0}}(f_1 Q_{bc} + f_2 Q_{c,1}) - \frac{\sigma_1\sigma_0}{\sigma_{m0}} Z_b + \sigma_0 Z_b\Big), \tag{L7}$$

with $f_{0,c}$ denoting the dropout spread, $f_{0.5,1}$ the 50% trimmed fraction in absence of dropout, $f_{b,1}$ the 50% trimmed fraction given dropout, and Z_b denoting the b quantile of a standard normal distribution.

We can distinguish three sources of bias as a consequence of strong MNAR assumption violation: underestimation of μ_{td0} (though $Q_{b,1}$, where dropout in the trimmed fraction results in a $b < 0.5$), overestimation of the mirrored comparator group distribution SD, σ_{0m}, and the contribution of Z_b, which, for $b < 0.5$, is now smaller than 0.

When the strong MNAR assumption is violated (missing values in the top half of the distribution), lower values will be introduced into the 50% trimmed fraction, which results in a lower 50% trimmed mean ($\mu_{td0} = \sigma_0 Q_{bd}$), and a comparatively larger trimmed fraction SD, which in turn results in an inflated mirrored full sample SD. With $\sigma_{0m} > \sigma_0$, $\frac{\sigma_1\sigma_0}{\sigma_{m0}} < \sigma_1$, and μ_{atd0} is biased further downwards. The remaining component of (L7), $\frac{\sigma_1\sigma_0}{\sigma_{m0}} Z_b + \sigma_0 Z_b$, will result in either a further bias downwards, for $\sigma_0 > \sigma_1$, or will partly mitigate it, for $\sigma_0 < \sigma_1$. Formulae for the mirrored full sample variance σ_{m0}, in presence and absence of dropout are derived in Section L.5.

L.2 Adjusted estimator bias on rescaling the comparator group, given strong MNAR violation in both intervention groups

When the strong MNAR assumption is violated in both treatment groups, two sources of bias can be distinguished. The first results from the biased estimate of the rescaled trimmed mean of the comparator group, μ_{atd0} (L4), and is given by $(\mu_{at0} - \mu_{atd0})$ (L5). The second results from the biased estimated of the trimmed mean of the intervention group, μ_{td1}, and is given by $(\mu_{td1} - \mu_{t1})$ (see Section F). We define the adjusted estimator bias on rescaling the comparator group:

$$\begin{aligned} B_{at0} &= (\mu_{at0} - \mu_{atd0}) + (\mu_{td1} - \mu_{t1}) \\ &= (\mu_{at0} - \mu_{atd0}) + B_{tSM1} \end{aligned} \tag{L8}$$

The component $(\mu_{at0} - \mu_{atd0})$ takes a negative value and was previously defined in L5 and L7. B_{tSM1} is the bias of the unadjusted estimator, previously defined in (F5), which takes a positive value, resulting from the underestimation of the intervention group trimmed mean (Appendix F). When the strong MNAR assumption is violated in the comparator group, additional violation of this assumption in the intervention group will mitigate the negative bias resulting from the underestimation of μ_{atd0} in two ways. Firstly, by introducing a positive bias component to counteract the negative one; secondly, by counteracting the overestimated mirrored comparator group SD, σ_{m0}, as the SD extrapolated from the 50% trimmed intervention group fraction is now also inflated. Let us denote the extrapolated intervention group SD by σ_{e1}, and write the total bias as

$$\begin{aligned} B_{at0} = -\sigma_1 Q_{0.5,1} - \Big(-\frac{\sigma_{e1}\sigma_0}{\sigma_{m0}}(f_1 Q_{bc} + f_2 Q_{c,1}) - \frac{\sigma_{e1}\sigma_0}{\sigma_{m0}} Z_b + \sigma_0 Z_b \Big) + \\ B_{tSM1}, \end{aligned} \tag{L9}$$

with $f_1 = \frac{f_{0.5,c}}{f_{0.5,1}}$, $f_2 = \frac{f_{c,1}}{f_{0.5,1}}$, B_{tSM1} obtained from (F5), and σ_{e1} and σ_{m0} calculated as detailed in Section L.5, from (L19), (L21) and (D1). When comparing (L9) to the bias defined in (L7), where the strong MNAR assumption is violated in the comparator group only, we note the following two differences. Firstly, σ_1 has been replaced by σ_{e1} in (L9), as the SD can no longer be estimated from the sample, and has to be calculated from the observed 50% trimmed intervention group fraction. Secondly, an additional bias componenent, B_{tSM1} is introduced, to account for the biased estimation of the unadjusted intervention group trimmed mean.

L.3 Adjusted estimator bias on rescaling the intervention group, given strong MNAR violation in the comparator group

Here we consider the adjusted TM estimate obtained on rescaling the intervention group, under assumption of normality and for 50% trimming. We define β_{at1} and β_{atd1}, with $\beta_{at1} = \mu_{at1} - \mu_{t0}$ an unbiased estimate of β_t, and $\beta_{atd1} = \mu_{atd1} - \mu_{t0d}$ the adjusted TM estimate under strong MNAR assumption violation in the

comparator group. The adjusted estimator bias for dropout in the comparator group, under intervention group rescaling is then given by:

$$\begin{aligned} B_{at1} &= \beta_{atd1} - \beta_{at1} \\ &= (\mu_{atd1} - \mu_{td0}) - (\mu_{at1} - \mu_{t0}) \\ &= \mu_{atd1} - \mu_{at1} + \mu_{td1} - \mu_{t0} \\ &= (\mu_{atd1} - \mu_{at1}) + B_{tSM0}, \end{aligned} \tag{L10}$$

with B_{tSM0} being the bias when violating the strong MNAR assumption in comparator group (F4), and μ_{at1} and μ_{atd1} defined analogously to μ_{at0} (L2) and μ_{atd0} (L4), with

$$\mu_{at1} = \frac{\mu_{t1} - \mu_1}{\sigma_1/\sigma_0} + \mu_1, \tag{L11}$$

and

$$\mu_{atd1} = \frac{\mu_{td1} - \mu_{m1}}{\sigma_{m1}/\sigma_{e0}} + \mu_{m1}. \tag{L12}$$

As the strong MNAR assumption is satisfied for the intervention group, $\mu_{td1} = \mu_{t1}$, $\mu_{m1} = \mu_1 = 0$, and $\sigma_{m1} = \sigma_1$, and (L12) reduces to

$$\mu_{atd1} = \frac{\mu_{t1}}{\sigma_1/\sigma_{e0}} = \frac{-\sigma_1 Q_{0,0.5}}{\sigma_1/\sigma_{e0}} = -\sigma_{e0} Q_{0,0.5}. \tag{L13}$$

The only source of bias affecting the adjusted intervention group trimmed mean, μ_{atd1}, is σ_{e0}, which results in a only a slight overestimation of the intervention group trimmed mean and a small positive bias (when compared to the bias resulting from rescaling the comparator group in the same scenario, see Section L.1). Then, with $\mu_{at1} = -\sigma_0 Q_{0.5,1}$, we write the adjusted estimator bias on rescaling the treatment group, for strong MNAR violation in both groups as

$$\begin{aligned} B_{at1} &= (-\sigma_{e0} Q_{0.5,1}) - (-\sigma_0 Q_{0.5,1}) + B_{tSM0} \\ &= (\sigma_0 - \sigma_{e0}) Q_{0.5,1} + B_{tSM0}, \end{aligned} \tag{L14}$$

with B_{tSM0} defined previously in (F4).

L.4 Adjusted estimator bias on rescaling the intervention group, given strong MNAR violation in the intervention group and optionally in the comparator group

Here, we consider a scenario where the strong MNAR assumption is violated in the intervention group, and we rescale the intervention group, to obtain β_{at1}. Then, with (L10), we define the adjusted estimator bias, in a manner analogous to (L7) in Section L.1. This bias, B_{at1}, is negative and given by:

$$B_{at1} = -\frac{\sigma_0\sigma_1}{\sigma_{m1}}(f_1 Q_{bc} + f_2 Q_{c,1}) - \frac{\sigma_0\sigma_1}{\sigma_{m1}} Z_b + \sigma_1 Z_b + \sigma_0 Q_{0.5.1}), \tag{L15}$$

with $f_1 = \frac{f_{0.5,c}}{f_{0.5,1}}$ and $f_2 = \frac{f_{c,1}}{f_{0.5,1}}$. In the event of strong MNAR violation in the comparator group, the additional component B_{tSM0} (F4) is included:

$$B_{at1} = -\frac{\sigma_{e0}\sigma_1}{\sigma_{m1}}(f_1 Q_{bc} + f_2 Q_{c,1}) - \frac{\sigma_{e0}\sigma_1}{\sigma_{m1}} Z_b + \sigma_1 Z_b + \sigma_0 Q_{0.5,1} + B_{tSM0}. \tag{L16}$$

On rescaling the comparator group for the first scenario, with dropout only in the intervention group, the adjusted estimator bias will, analogous to (L14), be given by

$$B_{at0} = (\sigma_{e1} - \sigma_1) Q_{0.5,1} + B_{tSM1}. \tag{L17}$$

L.5 Obtaining the mirrored full sample SD under strong MNAR assumption violation

As a result of strong MNAR violation, the SD of the mirrored distribution for a given group j, σ_{jm}, is overestimated ($\sigma_{jm} > \sigma_j$). The variance of the trimmed fractions, $f_{0.5,1}$ or $f_{b,1}$, can be written as a function of two non-overlapping sub-fractions, $f_{0.5,c}$ and $f_{c,1}$, using the underlying sample means, variances and fraction sizes (O'Neill, 2014):

$$\begin{aligned} \sigma^2_{0.5,1} &= \frac{1}{f_1 + f_2}\left[f_1 \sigma^2_{0.5,c} + f_2 \sigma^2_{c,1} + \frac{f_1 f_2}{f_1 + f_2}(\mu_{0.5,c} - \mu_{c,1})^2\right] \\ &= f_1 \sigma^2_{0.5,c} + f_2 \sigma^2_{c,1} + f_1 f_2 (\mu_{0.5,c} - \mu_{c,1})^2, \end{aligned} \tag{L18}$$

and equivalently for the fraction, $f_{b,1}$:

$$\sigma^2_{b,1} = f_1 \sigma^2_{bc} + f_2 \sigma^2_{c,1} + f_1 f_2 (\mu_{bc} - \mu_{c,1})^2, \tag{L19}$$

with $f_1 = \frac{f_{0.5,c}}{f_{0.5,1}}$, $f_2 = \frac{f_{c,1}}{f_{0.5,1}}$, and the variance of a given distribution fraction given by the variance of a truncated normal distribution, calculated from (D1).

It is clear that the fraction variance in presence of dropout, σ^2_{b1}, will exceed the variance in absence of dropout, $\sigma^2_{0.5,1}$, as the straightforward consequence of a larger fraction being evaluated for the former ($b < 0.5$). This is equally apparent from equations (L18) and (L19), with $\sigma^2_{bc} > \sigma^2_{0.5,c}$ and $|\mu_{bc} - \mu_{c,1}| > |\mu_{0.5,c} - \mu_{c,1}|$.

From the fraction variances, $\sigma^2_{0.5,1}$ and $\sigma^2_{b,1}$, the variance of the mirrored distribution, σ^2_{m0}, is easily obtained through equation (L18), now applied to the observed fraction, $f_{0.5,1}$, and the mirrored fraction, $f'_{0.5,1}$, with $f_1 = f_2 = 0.5$. In the absence of dropout, we then have:

$$\sigma^2_{mj} = \sigma^2_j = \sigma^2_{0.5,1} + 0.25(\mu_{0.5,1} - \mu'_{0.5,1})^2, \tag{L20}$$

while in the presence of dropout we obtain:

$$\sigma^2_{mj} = \sigma^2_{b,1} + 0.25(\mu_{b,1} - \mu'_{b,1})^2. \tag{L21}$$

The variance of the mirrored distribution in absence of dropout (L20) can also be calculated using normal distribution rules, as previously shown in (C3), with $\sigma_j^2 = \frac{\sigma_{0.5,1}^2}{(1-2/\pi)}$.

References

O'Neill B. Some useful moment results in sampling problems. *American Statistician.* 2014;68(4):282-296.

M Companion to Table 1: Examples of bias calculations

Here, we consider the simulation reported in Table 1 in the main text, and employ the bias formulae to calculate the CCA estimator bias, B_C, the location shift assumption bias, B_{tLS}, the strong MNAR assumption bias, B_{tSM}, and the adjusted TM estimator bias under comparator group rescaling, B_{at0}. For simplicity, let us consider a single scenario from Table 1.b in the main text, in which both assumptions are violated, with 20% dropout ($p_d = 0.2$) spread homogeneously across 75% of the comparator distribution, for a comparator SD, $\sigma_0 = 1.5$, and a intervention group SD, $\sigma_1 = 1$.

M.1 CCA bias

Consider the CCA bias on violation of the homogeneous (random) dropout assumption in the comparator group, defined in (H6) and equation 16 in the main text. Then, for $\sigma_0 = 1.5$, dropout proportion, $p_d = 0.2$, dropout spread, $f_{0,c} = 0.75$ with upper bound, $c = 0.75$, the bias is given by

$$\begin{aligned}
B_C = -\sigma_0 \Bigg[& \left(f_{0,c} - \frac{(f_{0,c} - p_d)}{(1-p_d)} \right) \frac{\phi\big(\Phi^{-1}(c)\big)}{c} + \\
& \left((1 - f_{0,c}) - \frac{(1-f_{0,c})}{(1-p_d)} \right) \frac{-\phi\big(\Phi^{-1}(c)\big)}{1-c} \Bigg] \\
= -1.5 \Bigg[& \left(0.75 - \frac{(0.75 - 0.2)}{(1-0.2)} \right) \frac{\phi\big(\Phi^{-1}(0.75)\big)}{0.75} + \\
& \left((1 - 0.75) - \frac{(1-0.75)}{(1-0.2)} \right) \frac{-\phi\big(\Phi^{-1}(0.75)\big)}{1-0.75} \Bigg] \\
= -1.5 & * 0.11 = -0.16.
\end{aligned} \tag{M1}$$

M.2 Location shift assumption bias

Consider the location shift assumption bias defined in (C5) and equation 7 in the main text. Then, for $\sigma_1 = 1$, $\sigma_0 = 1.5$ and trimming fraction, $p = 0.5$, the bias is given by

$$B_{tLS} = (\sigma_1 - \sigma_0)\frac{\phi\big(\Phi^{-1}(p)\big)}{1-p} = (1-1.5)\frac{\phi\big(\Phi^{-1}(0.5)\big)}{0.5} = -0.40, \tag{M2}$$

M.3 Strong MNAR assumption bias

For 20% dropout ($p_d = 0.2$), spread across 75% of the distribution ($f_{0,c} = 0.75$), we define $b = f_{0,c} - f_{bc}$, with f_{bc} calculated as in (E3):

$$f_{bc} = \frac{f_{0,c(0)} f_{0.5,c(0)}}{(f_{0,c(0)} - p_{d0})} = \frac{0.75 * 0.25}{0.75 - 0.2} = 0.34, \tag{M3}$$

giving $b = f_{0,c} - f_{bc} = 0.75 - 0.34 = 0.41$. Consider the comparator group component of the strong MNAR assumption bias from (F4) and equation (14) in the main text, with $-\sigma_0 Q_{ab}$ the truncated normal mean for a given fraction a to b, as in (E6). The bias is given by

$$\begin{aligned} B_{tSM0} &= -\frac{f_{0.5,c(0)}}{f_{0.5,1}}\sigma_0\big(Q_{0.5,c(0)} - Q_{bc(0)}\big) \\ &= \frac{0.25}{0.5} \times -1.5\Bigg(\frac{\phi\big(\Phi^{-1}(0.75)\big) - \phi\big(\Phi^{-1}(0.5)\big)}{0.75 - 0.5} - \\ &\frac{\phi\big(\Phi^{-1}(0.75)\big) - \phi\big(\Phi^{-1}(0.409)\big)}{0.75 - 0.409}\Bigg) \\ &= 1.5 \times 0.059 = 0.09. \end{aligned} \tag{M4}$$

M.4 Adjusted TM estimator bias

We now consider the adjusted estimator bias, for dropout in the comparator group only, when rescaling the comparator group. The bias (L7) is given by

$$B_{at0} = -\sigma_1 Q_{0.5,1} - \Big(-\frac{\sigma_1\sigma_0}{\sigma_{m0}}(f_1 Q_{bc} + f_2 Q_{c,1}) - \frac{\sigma_1\sigma_0}{\sigma_{m0}} Z_b + \sigma_0 Z_b\Big). \tag{M5}$$

Components $Q_{0.5,1}$ and $Q_{b,c}$ are truncated standard normal distribution means, with $c = 0.75$, and $b = 0.41$, from (M3), giving

$$Q_{bc} = \frac{\phi\big(\Phi^{-1}(0.75)\big) - \phi\big(\Phi^{-1}(0.41)\big)}{(0.75 - 0.41)} = -0.21, \tag{M6}$$

with $Q_{bd} = -0.80$ calculated in the same manner.

Treatment and comparator group SDs are known, with $\sigma_1 = 1$ and $\sigma_0 = 1.5$. We obtain σ^2_{mj} (L21) from $\sigma^2_{b,1}$ (L19), and the latter from σ^2_{bc} and $\sigma^2_{c,1}$, using (D1). Then, for $b = 0.41$, $c = 0.75$ and trimming fraction $p = 0.5$:

$$\begin{aligned}\sigma^2_{bc} &= \sigma^2_j \Big[1 - \frac{\Phi^{-1}(c)\phi\big(\Phi^{-1}(c)\big) - \Phi^{-1}(b)\phi\big(\Phi^{-1}(b)\big)}{c-p} - \\ &\Big(\frac{\phi\big(\Phi^{-1}(c)\big) - \phi\big(\Phi^{-1}(b)\big)}{c-p}\Big)^2\Big] \\ &= 1.5^2 \Big[1 - \frac{\Phi^{-1}(0.75)\phi\big(\Phi^{-1}(0.75)\big) - \Phi^{-1}0.41\phi\big(\Phi^{-1}0.41\big)}{0.75-p} - \\ &\Big(\frac{\phi\big(\Phi^{-1}(0.75)\big) - \phi\big(\Phi^{-1}0.41\big)}{0.75-0.5}\Big)^2\Big] \\ &= 0.15,\end{aligned} \tag{M7}$$

and, analogously, for $c = 0.75$, $\sigma^2_{c,1} = \sigma^2_{0.75,1} = 0.54$.

To obtain $\sigma^2_{b,1}$, we first define truncated means, μ_{bc} and $\mu_{c,1}$, using (E6), with Q_{bc} already defined in M6), giving

$$\mu_{bc} = -\sigma_0 Q_{bc} = -1.5 \times 0.21 = 0.31, \tag{M8}$$

and, in the same manner, $\mu_{c,1} = -\sigma_0 Q_{c,1} = -1.5 \times -1.27 = 1.91$.

Filling in (L19), with fractions $f_1 = (f_{0.5,c}/f_{0.5,1})$ and $f_1 = (f_{c,1}/f_{0.5,1})$, we obtain

$$\begin{aligned}\sigma^2_{b,1} &= f_1\sigma^2_{bc} + f_2\sigma^2_{c,1} + f_1 f_2(\mu_{bc} - \mu_{c,1})^2 \\ &= 0.5 \times 0.15 + 0.5 \times 0.54 + 0.25(0.31 - 1.91)^2 \\ &= 0.98.\end{aligned} \tag{M9}$$

To obtain the mirrored comparator distribution mean, σ^2_{m0}, we first define μ_{bd} as the sum of weighted fraction means, with $\mu_{b,1} = f1\mu_{bc} + f2\mu_{c,1} = 0.5 \times 0.31 + 0.5 \times 1.91 = 1.11$, and the mirrored mean, $\mu'_{b,1}$, with $\mu'_{b,1} = -\mu_{b,1} + 2\sigma_0 Z_b = -1.11 + 2 \times -0.23 = -1.80$, where $Z_b = -0.23$ is the normal quantile corresponding to $b = 0.41$ and also the mean of the mirrored distribution, μ_{m0}.

Filling in (L21), we obtain:

$$\begin{aligned}\sigma^2_{mj} &= \sigma^2_{b,1} + 0.25(\mu_{b,1} - \mu'_{b,1})^2 \\ &= 0.98 + 0.25(1.11 - -1.80)^2 \\ &= 3.10,\end{aligned} \tag{M10}$$

with $\sigma_{mj} = \sqrt{3.10} = 1.76$. We now fill in (M6) and obtain the adjusted estimator bias under comparator group rescaling and comparator group strong MNAR violation:

$$
\begin{aligned}
B_{at0} &= -\sigma_1 Q_{bd} - \Big(-\frac{\sigma_1\sigma_0}{\sigma_{m0}}(f_1 Q_{bc} + f_2 Q_{c,1}) - \frac{\sigma_1\sigma_0}{\sigma_{m0}} Z_b + \sigma_0 Z_b \Big) \\
&= -1 \times -0.8 - \Big(-\frac{1 \times 1.5}{1.76}(0.5 \times -0.21 + 0.5 \times -1.27) - \\
&\frac{1 \times 1.5}{1.76} - 0.23 + 1.5 \times -0.23 \Big) \\
&= 0.32.
\end{aligned} \tag{M11}
$$

N Trade-off of location shift bias and strong MNAR bias under different trimming proportions

Let us once more consider the unadjusted TM estimator bias components, under dropout in the comparator group. From (C5) we have the location shift assumption bias:

$$
B_{tLS} = (\sigma_1 - \sigma_0)\frac{\phi\big(\Phi^{-1}(p)\big)}{1-p}, \tag{N1}
$$

and from (F4) the strong MNAR assumption bias:

$$
B_{tSM0} = -\frac{f_{0.5,c(0)}}{f_{0.5,1}}\sigma_0(Q_{0.5,c(0)} - Q_{bc(0)}), \tag{N2}
$$

with $b_0 = f_{0,c(0)} - f_{bc(0)}$, and

$$
f_{bc(0)} = \frac{f_{0,c(0)} f_{bc(0)}}{(f_{0,c(0)} - p_{d0})}. \tag{N3}
$$

In the main text, we primarily consider fixed 50% trimming. From (N1) and (N2, N3) we observe that B_{tLS} and B_{tSM}, respectively, are also a function of the specified trimming proportion, p. More specifically, a larger p will increase B_{tLS}, but decrease B_{tSM}. This bias trade-off is illustrated in Supplementary Figure S4, which shows the two bias components against the trimming fraction, p, for a scenario with 20% comparator group dropout ($p_d = 0.2$), spread across 75% of the distribution ($f_{ac} = 0.75$).

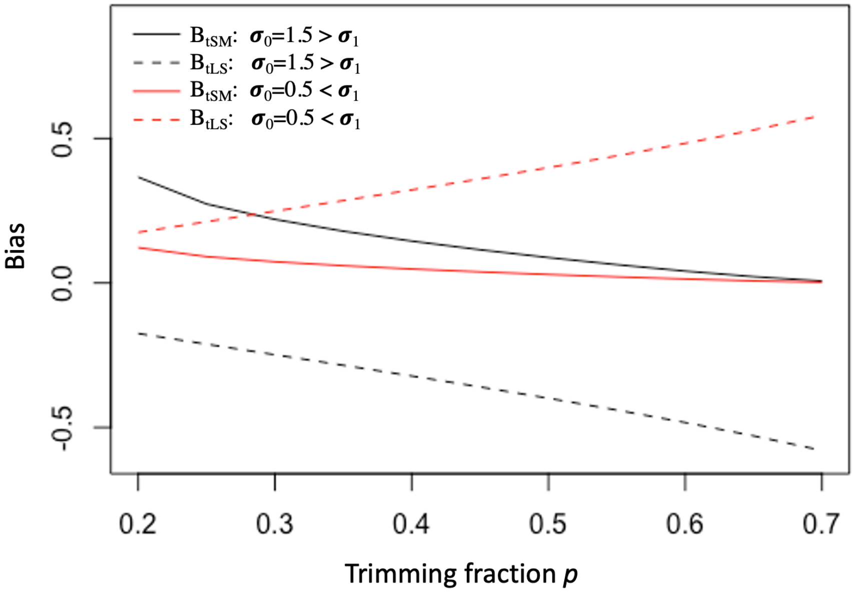

Figure S4: Location shift assumption bias B_{tLS} (dashed line) and strong MNAR assumption bias B_{tSM} (solid line) across different trimming proportions p, under 20% comparator group dropout spread across 75% of the distribution ($f_{ac} = 0.75$), for comparator group SDs $\sigma_0 = 0.5$ (red) and $\sigma_0 = 1.5$ (black), and intervention group SD $\sigma_1 = 1$

O R code for obtaining unadjusted and unadjusted TM estimates

TM.sim.func is a function for simulating data from an RCT and performing an unadjusted and adjusted TM analysis under comparator group dropout and for 50% trimming. *TM.sim.func* was used to generate the results of Table 1 in the main text. The function takes the following arguments:

- comparator group dropout proportion (*plD*)
- intervention group SD (*TRSD*)
- comparator group SD (*PLSD*)
- treatment effect size (*effTR*)
- sample size (n)
- dropout spread (e.g., *LOW* restricts all dropout to the lowest *plD*×100%)
- optional permutation function (when specifying *perm="YES"*, to obtain P value and 95% CI for unadjusted TM estimator

The function returns the following output:

- Specified treatment effect size (*effTR*)
- CCA estimate (*beta_c*)
- Unadjusted TM estimate (*beta_t*)
- Adjusted TM estimate, when rescaling the intervention group (*beta_t_resc_TR*)
- Adjusted TM estimate, when rescaling the comparator group (*beta_t_resc_PL*)

- The P-value and 95% confidence interval for the unadjusted TM estimate (*out_extra*)

```
TM.sim.func <- function(plD=0.2, TRSD=1, PLSD=1, n=10000,
                        effTR=0.5, opt="LOW", perm=FALSE,
                        a0 = 0, seed = NULL){
  if (!is.null(seed)) set.seed(seed)

  # patients randomized to treatment
  Tr <- rbinom(n,1,0.5)
  Tr <- sort(Tr, decreasing=FALSE)

  # generating normally distributed outcomes in intervention group,
  #with treatment effect effTR
  Yfin.mean.TR <- a0 + effTR
  Yfin.TR <- rnorm(length(which(Tr==1)),Yfin.mean.TR, TRSD)

  # generating normally distributed outcomes in comparator group
  Yfin.mean.PLAC <- a0
  Yfin.PLAC <- rnorm(length(which(Tr==0)), Yfin.mean.PLAC, PLSD)

  # joint outcome vector
  Yfin.TR <- sort(Yfin.TR)
  Yfin.PLAC <- sort(Yfin.PLAC)
  Yfin <- vector("numeric", n)
  Yfin[which(Tr==1)] <- Yfin.TR
  Yfin[which(Tr==0)] <- Yfin.PLAC


  # Number of dropouts (with plD specifying the dropout proportion)
  s.set <- round(plD*length(which(Tr==0)),0)


  # Generating dropout for four scenarios

    # Strict lower value dropout
  if(opt=="LOW"){
    pl.out <- (seq(1,plD*length(which(Tr==0)),1))
    }
    # Dropout in bottom 50% of the distribution
  if(opt=="LOWSCAT"){
    pl.out <- sample(seq(1,length(which(Tr==0))/2),s.set)
    }
    # Dropout in bottom 75% of the distribution
  if(opt=="SCAT"){
    pl.out <- sample(seq(1,round(length(which(Tr==0))*0.75),0),s.set)}

    # Dropout across 100% of the distribution
  if(opt=="ALL"){
    pl.out <- sample(seq(1,round(length(which(Tr==0))*0.75),0),s.set)}


  # Generating dropout indicator
  C.outs <- rep(1,n)
```

```r
C.outs[c(pl.out)] <- 0

# Generating intervention group data with a column for outcome when
# fully observed (Yfin) and outcome after dropout (V4), where
# dropout are assigned a value (-Inf) lower than the smallest
# observed outcome. In this function there is no option for
# specifying intervention group dropout, so Yfin=V4 for the
# intervention group
dat.pT <- as.data.frame(cbind(IDs=seq(1,length(which(Tr==1)),1),
                              Yfin=Yfin[which(Tr==1)],
                              Tr=c(rep(1,length(which(Tr==1)))),
                              outs=C.outs[which(Tr==1)], V4=NA))
dat.pT$V4[which(dat.pT$outs==1)] <- dat.pT$Yfin[which(dat.pT$outs==1)]
dat.pT$V4[which(dat.pT$outs==0)] <- -Inf

# Generating comparator group data with a column for outcome when
# fully observed (Yfin) and outcome after dropout (V4), where
# dropouts are assigned a value (-Inf) lower than the smallest
# observed outcome.
dat.pP <- as.data.frame(cbind(IDs=seq(length(which(Tr==1))+1,
                                      length(Tr),1),
                              Yfin=Yfin[which(Tr==0)],
                              Tr=c(rep(0,length(which(Tr==0)))),
                              outs=C.outs[which(Tr==0)], V4=NA))

dat.pP$V4[which(dat.pP$outs==1)] <- dat.pP$Yfin[which(dat.pP$outs==1)]
dat.pP$V4[which(dat.pP$outs==0)] <- -Inf

# sorting outcomes per intervention group and 50% trimming
dat.ex.tr.sort <- dat.pT[order(dat.pT$V4),]
dat.ex.plac.sort <- dat.pP[order(dat.pP$V4),]

trim.frac.tr <- round(0.5*nrow(dat.ex.tr.sort),0)
trim.frac.plac <- round(0.5*nrow(dat.ex.plac.sort),0)

dat.ex.tr.trim <- dat.ex.tr.sort[-(1:trim.frac.tr),]
dat.ex.plac.trim <- dat.ex.plac.sort[-(1:trim.frac.plac),]

# CCA estimate
compdat <- rbind(dat.pT, dat.pP)
compdat <- compdat[which(compdat$outs==1),]
beta_c <- summary(lm(Yfin~Tr, compdat))$coefficients[2,1]

# Unadjusted TM estimate
beta_t <- mean(dat.ex.tr.trim$Yfin)-mean(dat.ex.plac.trim$Yfin)


# Adjusted TM estimate, obtained by rescaling the intervention group
  # extrapolating full sample SD from comparator group trimmed fraction
sd.tr.PL <- sqrt((sd(dat.ex.plac.trim$Yfin)^2)/(1-2/pi))
  # unscaled outcome
x1 <- dat.ex.tr.trim$Yfin
  # mirrored distribution of unscaled outcome
x2 <- min(x1) - (abs((min(x1)-x1)))
```

```r
  # pooled distribution, after mirroring
x3a <- c(x1,x2)
  # rescaling mirrored intervention group distribution
x3 <- x3a - mean(x3a)
x4 <- x3/(sd(x3)/sd.tr.PL)
x4 <- x4 - mean(x4)
x5 <- x4 + mean(x3a)
  # obtaining original, now rescaled values
x6 <- x5[1:length(x1)]

  # replacing unscaled values in dataframe with rescaled ones
dat.ex.tr.trim2 <- dat.ex.tr.trim
dat.ex.tr.trim2$Yfin <- x6

beta_t_resc_TR <- mean(dat.ex.tr.trim2$Yfin)-
                  mean(dat.ex.plac.trim$Yfin)


# Adjusted TM estimate, obtained by rescaling the comparator group,
# using the SD of the fully observed intervention group
sd.tr.TR <- sd(dat.ex.tr.sort$Yfin)
# unscaled outcome
x1 <- dat.ex.plac.trim$Yfin
# mirrored distribution of unscaled outcome
x2 <- min(x1) - (abs((min(x1)-x1)))
# pooled distribution, after mirroring
x3a <- c(x1,x2)
# rescaling mirrored intervention group distribution
x3 <- x3a - mean(x3a)
x4 <- x3/(sd(x3)/sd.tr.TR)
x4 <- x4 - mean(x4)
x5 <- x4 + mean(x3a)
# obtaining original, now rescaled values
x6 <- x5[1:length(x1)]
# replacing unscaled values in dataframe with rescaled ones
dat.ex.plac.trim2 <- dat.ex.plac.trim
dat.ex.plac.trim2$Yfin <- x6

beta_t_resc_PL <- mean(dat.ex.tr.trim$Yfin)-
                      mean(dat.ex.plac.trim2$Yfin)

# output with true treatment effect, CCA estimate, TM estimate,
# adjusted TM estimate under intervention group rescaling,
# adjusted TM estimate under comparator group rescaling
out <- c(effTR, beta_c, beta_t, beta_t_resc_TR, beta_t_resc_PL)
out.extra <- c(0,0,0)

# permutation function for confidence intervals and P-value
# unadjusted TM estimator
perm.ff <- function(dat.trim){
  new.tr <- sample(dat.trim$Tr, replace=FALSE)
  perm.est <- summary(lm(dat.trim$Yfin~new.tr))$coefficients[2,1]
  return(perm.est)
}
```

```
  if(perm==TRUE){
    perm.testing <- replicate(1000, perm.ff(dat.trim))
      # obtaining P value
    Pval <- (length(perm.testing)-sum(beta_t>perm.testing))/
                    length(perm.testing)
      # obtaining confidence interval
    sd.perm <- sd(perm.testing)
    conf.int <- c(beta_t-sd.perm*1.96, beta_t+sd.perm*1.96)
    out.extra <- c(Pval, conf.int)
  }



  # output with true treatment effect, CCA estimate, TM estimate,
  # Adjusted TM estimate under intervention group rescaling, Adjusted
  # TM estimate under comparator group rescaling, TM estimate P value,
  # and TM estimate 95% CI
  if(perm==TRUE){
    out <- c(effTR, beta_c, beta_t, beta_t_resc_TR, beta_t_resc_PL,
                out.extra)
  }

  return(out)
}
```

P Application using the CoBalT RCT: BDI-II score distributions across time and intervention groups

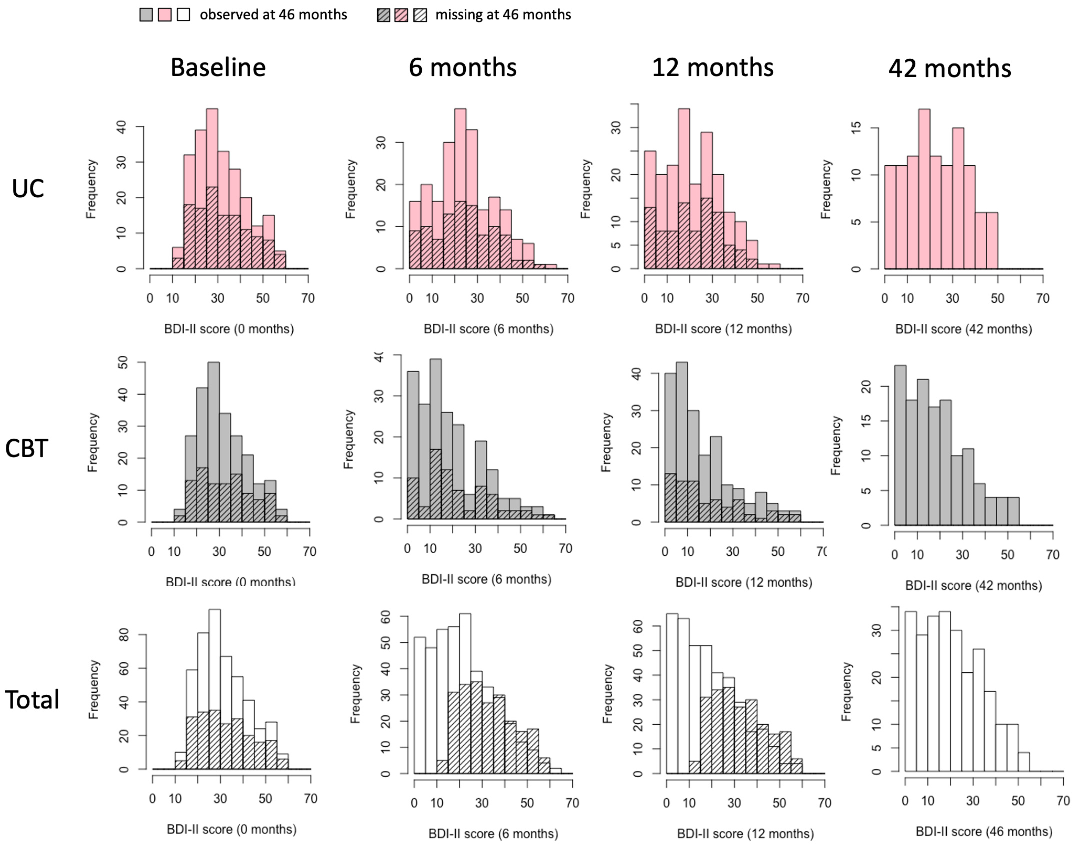

Figure S5: BDI-II score distributions at baseline, 6, 12 and 42 months for the usual care comparator group (UC, pink), the CBT intervention group (CBT, gray), and both groups jointly (Total, white). Outcome values missing at 42 months are indicated with a diagonal stripe pattern for each of the preceding time points.

Q Inferring the full sample SD from the SD observed under dropout, and calculating bias for the CoBalT application (R code)

For a given dropout scenario, the TM estimator bias can be quantified using the formulae detailed in Sections 2.3 and 2.4 of the main text (see also Appendices C-F). Both the location shift assumption bias and the strong MNAR bias are a function of the full sample SDs, which remain unobserved in the presence of dropout. Under the assumption of normally distributed outcomes these can, however, for a given dropout mechanism, be inferred from the observed SDs. The specified dropout mechanism has implications for the underlying full sample SDs. Consider, for example, the scenario in Figure 2.2 of the main text, where we have 41.9% fully directional dropout in the treatment (CBT) group and 52.3% entirely homogeneous dropout in the comparator (UC) group, which satisfies and violates the strong MNAR assumption in the treatment and comparator group, respectively. While the observed comparator group SD, under homogeneous dropout, is representative of the full sample SD, the observed intervention group SD, under assumption of highest value dropout, is the SD of a partial distribution. Under assumption of normality, we can consider this the SD of a normal distribution truncated at 41.9%, and infer the corresponding full sample SD.

Consider the variance of a truncated normal distribution, σ^2_{ab},for a given fraction of the distribution, $f_{a,b}$ with left-truncation at a and right-truncation at b (see also (D1), Appendix D):

$$\sigma^2_{ab} = \sigma^2\Big[1 - \frac{\Phi^{-1}(b)\phi\big(\Phi^{-1}(b)\big) - \Phi^{-1}(a)\phi\big(\Phi^{-1}(a)\big)}{b-a} - \Big(\frac{\phi\big(\Phi^{-1}(b)\big) - \phi\big(\Phi^{-1}(a)\big)}{b-a}\Big)^2\Big], \tag{Q1}$$

with corresponding truncated mean (see also (C2), Appendix C):

$$\mu_{ab} = \mu - \sigma\frac{\phi\big(\Phi^{-1}(b)\big) - \phi\big(\Phi^{-1}(a)\big)}{b-a}. \tag{Q2}$$

We consider the case of higher value dropout, as in Figure 2.2 in the main text, and denote the fraction of the outcome distribution unaffected by dropout $f_{0,c}$, and the fraction of the distribution affected by dropout $f_{c,1}$. Previously, for lower value dropout, we used $f_{0,c}$ to denote the fraction with dropout, while here, as we look at higher value dropout, we use $f_{c,1}$ (note that the location of dropout, be it towards the top or bottom of distribution, does not impact the observed or full sample SD). The corresponding variances, $\sigma^2_{0,c}$ and $\sigma^2_{c,1}$, and truncated means, $\mu_{0,c}$ and $\mu_{c,1}$, can then be used to obtain the total variance. Note that the variance of the fraction affected by dropout, $\sigma^2_{c,1}$, under the assumption of homogeneous dropout in this fraction, is equal to its variance in absence of dropout.

Let $f_1 = \frac{f_{c,1}-p_d}{1-p_{d0}}$ and $f_2 = \frac{f_{0,c}}{1-p_{d0}}$ denote the proportions of observations, after dropout, in $f_{c,1}$, and $f_{0,c}$, respectively. The observed variance, after dropout, can be written as a function of the variances and means of the two fractions (see also (L18), Appendix L.5):

$$\sigma^2_o = f_1\sigma^2_{0,c} + f_2\sigma^2_{c,1} + f_1 f_2(\mu_{0,c} - \mu_{c,1})^2, \tag{Q3}$$

Assuming, without loss of generality, a 0-centered distribution, and using (Q1) and (Q2), we write (Q3) as a function of the full variance, σ^2:

$$\begin{aligned}\sigma^2_o = {} & f_1\sigma^2\Big[1 - \frac{\Phi^{-1}(c)\phi\big(\Phi^{-1}(c)\big)}{c} - \Big(\frac{\phi\big(\Phi^{-1}(c)\big)}{c}\Big)^2\Big] + \\ & f_2\sigma^2\Big[1 + \frac{\Phi^{-1}(c)\phi\big(\Phi^{-1}(c)\big)}{1-c} - \Big(\frac{\phi\big(\Phi^{-1}(c)\big)}{1-c}\Big)^2\Big] + \\ & f_1 f_2(-\sigma\frac{\phi\big(\Phi^{-1}(c)\big)}{c} - \sigma\frac{\phi\big(\Phi^{-1}(c)\big)}{1-c})^2.\end{aligned} \tag{Q4}$$

By filling in the observed variance, specifying the dropout spread and proportion, we can solve for σ and infer the unobserved, full sample SD. Below, R code is given for the implementation of this. Additionally, code is provided for functions that calculate the location shift assumption bias and strong MNAR assumption bias. These are used to calculate the expected bias for the TM estimate of the CBT treatment effect in the CoBalT data, for three different dropout mechanisms.

```r
# function for calculating full sample SD from observed
# variance (obs.var), dropout spread (DS) and dropout
# proportion (dr), under assumption of normality implements
# equation (Q4)
# this function can be used for dropout on either side of
# the distribution. If dropout is in the fraction f_{0,c},
# on the lower end of the distribution, we use DS=c, to
# calculate the appropriate dropout spread. If dropout is on
# the higher end of the distribution, in the fraction
# $f_{c,1}$, we use DS=1-c.

SD.func.extr <- function(DS, dr, obs.var){

  c <- DS
  fr1 <- (c-dr)/(1-dr)
  fr2 <- (1-c)/(1-dr)

  A <- (1- ((qnorm(c)*dnorm(qnorm(c)))/(c)) -
           ((dnorm(qnorm(c))/(c)))^2)
  B <- (1- ((-qnorm(c)*dnorm(qnorm(c)))/(1-c))-
           ((-dnorm(qnorm(c))/(1-c)))^2)
  C <- -(dnorm(qnorm(c)))/(c)
  D <- (dnorm(qnorm(c)))/(1-c)

  fsd <- function(sigf) {((fr1*A*sigf^2 + fr2*B*sigf^2 +
                        fr1*fr2*(C*sigf-D*sigf)^2))-
                          obs.var}

  fullSD <- uniroot(fsd, lower=0.1, upper=30)$root
  return(fullSD)}


# Function for calculating the location shift assumption bias
# for a given intervention group SD (TRSD) and comparator group SD
# (PLSD), for a specified trimming fraction (trim.frac)
# Implements equation (B5) in Appendix (B)
LSA.bias <- function(TRSD, PLSD, trim.frac){
  -(TRSD - PLSD) * (dnorm(qnorm(1-trim.frac))-dnorm(qnorm(0)))/
                       (1-trim.frac)}


# Function for calculating the strong MNAR assumption bias for a
# given intervention group (group="TR or group="PL), from the full
# sample SD (SD), the dropout spread (DSP), the dropout proportion
# (plD) and the trimming fraction (trimf).
# implements equations (E4) and (E5) in Appendix E
bias.drop <- function(SD=1,DSP=0.9, plD=0.2, trimf=0.55,
                group="TR"){

  DS <- 1-DSP
  calc.frac <- ((1-DS) * ((1-DS)-trimf) / ((1-DS)-plD))

  if (DSP <= trimf){
    calc.frac <- 0
```

```
  }
  calc.frac

  b.prop <- 1-trimf
  c.prop <- DS

  bst.prop <- DS+calc.frac
  a <- qnorm(DS+calc.frac,0,1)
  b <- qnorm(DS,0,1)
  a1 <- qnorm(1-trimf,0,1)

  F1 <- (((1-trimf)-DS)/(1-trimf))

  if(DSP<=trimf){
    a <- qnorm(1-trimf,0,1)
    b <- qnorm(0,0,1)
    a1 <- qnorm(1-trimf,0,1)
    F1 <- 1
  }

  elPL <- ((((dnorm(a1)-dnorm(b))/
                   (pnorm(a1)-pnorm(b)))*-SD)-
        ((dnorm(a)-dnorm(b))/
                (pnorm(a)-pnorm(b))*-SD)) *F1
  elTR <- (((dnorm(a)-dnorm(b))/
                (pnorm(a)-pnorm(b))*-SD) -
        ((((dnorm(a1)-dnorm(b))/
                (pnorm(a1)-pnorm(b)))*-SD))) *F1

  if(group=="TR"){
    el <- elTR
  } else { if (group=="PL"){
    el <- elPL}
  }

  if(group!="PL" & group!="TR"){
    el <- NA}

  el

  return(el)}

# Calculating expected bias for the CBT treatment effect
# estimate in CoBalT data, under 53.3% trimming, for three
# different dropout scenarios.

# comparator (UC) group with homogeneous dropout, treatment
# (CBT) group with completely directional dropout in
# upper 41.9%
  # comparator (UC) group SD
SD.PL.1 <- SD.func.extr(DS=0.999, dr=0.533,
            obs.var=13.2^2) # 13.12
  # treatment (CBT) group SD
SD.TR.Dir <- SD.func.extr(DS=0.419, dr=0.419,
```

```r
            obs.var=13.8^2) # 21.54
  # Location shift assumption bias
LS_bias_a <- LSA.bias(SD.TR.Dir, SD.PL.1, 0.533) # -7.17
  # Strong MNAR assumption bias in comparator (UC) group
SM_bias_PL_a <- bias.drop(SD=SD.PL.semi_Hom, DSP=1, plD=0.533,
                          trimf=0.533, group="PL") #-10.36
  # Total bias
tot_bias_a <- LS_bias_a+SM_bias_PL_a # -17.53


# comparator (UC) group with homogeneous dropout, treatment
# (CBT) group with completely directional dropout in upper 60%
  # comparator (UC) group SD
SD.PL.1 <- SD.func.extr(DS=0.9999, dr=0.533,
                       obs.var=13.2^2)
  # treatment (CBT) group SD
SD.TR.semi_Dir1 <- SD.func.extr(DS=0.6, dr=0.419,
                               obs.var=13.8^2)
  # Location shift assumption bias
LS_bias_b <- LSA.bias(SD.TR.semi_Dir1, SD.PL.1, 0.533) # -1.14
  # Strong MNAR assumption bias in comparator (UC) group
SM_bias_PL_b <- bias.drop(SD=SD.PL.semi_Hom, DSP=1, plD=0.533,
                        trimf=0.533, group="PL") #-10.36
  # Strong MNAR assumption bias in treatment (CBT) group
SM_bias_TR_b <- bias.drop(SD=SD.TR.semi_Dir2, DSP=0.6,
                 plD=0.419,trimf=0.533,group="TR") # 0.41
  # total bias
tot_bias_b <- LS_bias_b+SM_bias_PL_b+SM_bias_TR_b #-11.09

# comparator (UC) group with mostly homogeneous dropout in
# upper 80% of the distribution, and treatment (CBT) group with
# mostly directional dropout in upper 60%
  # comparator (UC) group SD
SD.PL.semi_Hom <- SD.func.extr(DS=0.8, dr=0.533,
                       obs.var=13.2^2)
  # treatment (CBT) group SD
SD.TR.semi_Dir2 <- SD.func.extr(DS=0.6, dr=0.419,
                       obs.var=13.8^2)
  # Location shift assumption bias
LS_bias_c <- LSA.bias(SD.TR.semi_Dir2, SD.PL.semi_Hom, 0.533) # -2.01
  # Strong MNAR assumption bias in comparator (UC) group
SM_bias_PL_c <- bias.drop(SD=SD.PL.semi_Hom, DSP=0.8, plD=0.533,
                        trimf=0.533,group="PL") #-5.50
  # Strong MNAR assumption bias in treatment (CBT) group
SM_bias_TR_c <- bias.drop(SD=SD.TR.semi_Dir2, DSP=0.6, plD=0.419,
                        trimf=0.533,group="TR") # 0.41
  # Total bias
tot_bias_c <- LS_bias_c+SM_bias_PL_c+SM_bias_TR_c #-7.10
```
